# Regulation of host gene expression by HIV-1 TAR microRNAs

**DOI:** 10.1186/1742-4690-10-86

**Published:** 2013-08-12

**Authors:** Dominique L Ouellet, Jimmy Vigneault-Edwards, Kevin Létourneau, Lise-Andrée Gobeil, Isabelle Plante, John C Burnett, John J Rossi, Patrick Provost

**Affiliations:** 1 Department of Molecular and Cellular Biology, Beckman Research Institute at City of Hope, 1500 E Duarte Road, Duarte, CA 91010, USA; 2 CHUQ Research Center/CHUL, 2705 Blvd Laurier, Quebec, QC G1V 4G2, Canada; 3 Faculty of Medicine, Université Laval, Quebec, QC G1V 0A6, Canada

**Keywords:** HIV-1, TAR microRNAs, Apoptosis, Caspase 8, Ikaros, Aiolos, Nucleophosmin (NPM)/B23

## Abstract

**Background:**

The transactivating response (TAR) element of human immunodeficiency virus type 1 (HIV-1) is the source of two functional microRNAs (miRNAs), miR-TAR-5p and miR-TAR-3p. The objective of this study was to characterize the post-transcriptional regulation of host messenger RNAs (mRNAs) relevant to HIV-1 pathogenesis by HIV-1 TAR miRNAs.

**Results:**

We demonstrated that TAR miRNAs derived from HIV-1 can incorporate into host effector Argonaute protein complexes, which is required if these miRNAs are to regulate host mRNA expression. Bioinformatic predictions and reporter gene activity assays identified regulatory elements complementary and responsive to miR-TAR-5p and miR-TAR-3p in the 3’ untranslated region (UTR) of several candidate genes involved in apoptosis and cell survival. These include Caspase 8, Aiolos, Ikaros and Nucleophosmin (NPM)/B23. Analyses of Jurkat cells that stably expressed HIV-1 TAR or contained a full-length latent HIV provirus suggested that HIV-1 TAR miRNAs could regulate the expression of genes in T cells that affect the balance between apoptosis and cell survival.

**Conclusions:**

HIV-1 TAR miRNAs may contribute to the replication cycle and pathogenesis of HIV-1, by regulating host genes involved in the intricate balance between apoptosis and infected cell, to induce conditions that promote HIV-1 propagation and survival.

## Background

Endogenous miRNAs are generally exported as ~60 to 70 nucleotide (nt) miRNA precursors [1] from the nucleus to the cytoplasm, where they are processed by the ribonuclease III Dicer-TAR RNA-binding protein (TRBP) complex [2-4] into ~21-24 nt mature miRNA products. The Dicer-TRBP complex recruits the Argonaute (Ago) enzyme, which is loaded with the duplex RNA [5-8]. After strand dissociation, Ago can act on messenger RNAs (mRNAs), that have complementary sequences to the loaded guide miRNA, and block protein expression by repressing mRNA translation or inducing mRNA degradation [9].

The HIV-1 retrovirus has an RNA genome that is converted into complementary DNA (cDNA) by the viral reverse transcriptase and subsequently into double stranded DNA (dsDNA), prior its integration into the human host genome. Viral transcription is initiated from the 5’ long terminal repeat (LTR) promoter by the cellular RNA polymerase II. Unspliced and spliced RNAs are further exported to the cytoplasm by the HIV-1 Rev protein, to be respectively packaged into virion particles or translated into HIV proteins. Viral transcription is enhanced when the HIV Tat protein binds to the TAR, a structured RNA element present at the 5’ end of all RNA transcripts derived from the virus [10-12]. In the absence of Tat, transcription is aborted and short non-polyadenylated RNA transcripts encoding the first ~60 nt of the HIV-1 RNA genome, containing the 5’ end TAR element, are produced [13-15]. TAR RNA structure analysis have shown some evolutionary links between members of different groups [16] and a high degree of nucleotide sequence conservation between subtypes [17]. HIV-1 TAR RNA structure is highly conserved among different biological states [18,19] and shows a high resistance to mutations when RNA interference is directed against the viral TAR hairpin [20].

We previously reported TAR from the 5’ end LTR, which resembles endogenous pre-miRNAs, as a source of small non-coding regulatory RNAs. The TAR hairpin motif is also present within the 3’LTR of full-length HIV transcripts [17,21,22] but the adjacent polyadenylation signals that are used exclusively within the 3’ LTR context may render the site less prone to cleavage by Dicer. We identified and characterized two miRNAs, miR-TAR-5p and miR-TAR-3p, that are derived from this element [23]. Similar miRNA sequences or small RNAs derived from HIV-1 have also been reported by others [24-26]. Furthermore, cellular mRNAs that have sequences complementary to HIV-1 miRNAs in their 3’ UTRs, and therefore, are potential regulatory targets of these viral miRNAs, have been identified [27]. Indeed, a role has been suggested for HIV-1 miRNAs in transcriptional repression mediated either by the HIV-1 miR-N367 [26] or TAR miRNAs binding to the LTR promoter [24,28]. In addition, Klase et al. showed that TAR miRNA expression in TAR-expressing cells conferred resistance to apoptosis, and suggested that both the ERCC1, and IER3 genes, found to be targeted by TAR miRNAs in their assays, might be link to the observed effect [29]. Similarly, a viral miRNA, miR-BART-5 derived from the Epstein-Barr virus (EBV) [30], targets the mRNA of the host cell p53 up-regulator of apoptosis (PUMA), to reduce the likelihood of the EBV-infected cells from undergoing apoptosis-induced cell death [31]. Since TAR miRNAs have been recently detected in exosomes released from HIV-1 infected cells, their distributions within the non-infected cell population might be enhanced [32].

HIV-1 infects CD4^+^ T cells and apoptosis is known to be a significant cause of HIV-1-infected, CD4^+^ T cell death [33]. Many HIV-1 proteins are reported to regulate apoptosis by interfering with or promoting specific pro- or anti-apoptotic cellular components [33]. Although HIV-1 can induce the apoptosis of infected CD4^+^ T lymphocytes by distinct pathways, i.e. “from without”, “from within” or *in trans*[33,34], the precise mechanisms remain uncertain [35-37].

In this study, we examined the regulatory properties of the TAR miRNAs to identify cellular targets involved in the pathogenesis of HIV-1. We found that HIV-1 TAR miRNAs target and modulate the expression of host cell mRNAs for Caspase 8, an initiator protein of the extrinsic apoptosis pathway [38], the Aiolos and Ikaros, transcription factors involved in hematopoiesis, immunity and cell fate [39], and Nucleophosmin (NPM)/B23, a multifunctional nucleolar protein [40]. Our findings suggest that HIV-1 TAR miRNA-mediated regulation of these genes could shift the delicate balance between apoptosis and the survival of infected cells, thereby creating a cellular environment favourable to the replication and persistence of the virus.

## Results

### TAR miRNAs are incorporated into Argonaute protein complexes

**Figure 1:**
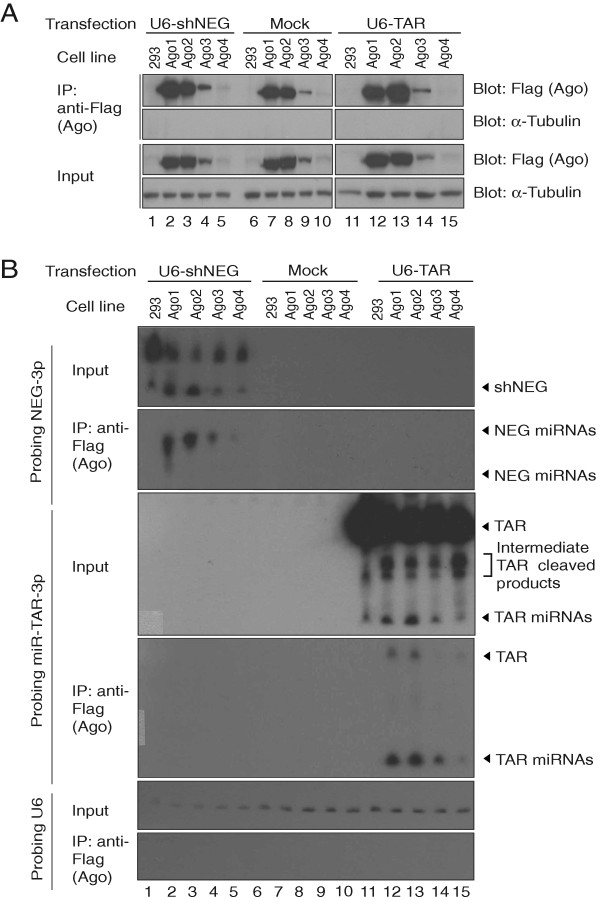
**HIV-1 TAR miRNAs are loaded into Argonaute complexes.** Stable HEK 293 cells expressing Flag-Argonaute 1–4 proteins were transfected with either U6-shNEG or U6-TAR or not (mock) for 48 hours. Protein samples were collected (input) and immunoprecipitated (IP) by an anti-Flag antibody. After the last wash, ¼ of the IP sample was kept for protein analysis and ¾ for RNA analysis. All solutions were prepared with DNA/RNA nuclease free water and contain protease and RNAse inhibitors. **A)** Western blot analysis of Argonaute (Ago) proteins from transfected samples. Input and IP samples were separated on a 10% polyacrylamide gel and immunoblotted with anti-Flag antibody. **B)** Northern blot of miRNAs expressed from U6-shNEG or U6-TAR transfected cells using probes against NEG-3p or miR-TAR-3p miRNAs. The probe against U6 RNA was used as a loading control for input and IP samples.

Argonaute proteins are the core components of the RNA silencing complexes (RISC) and exert their repressive effects on specific mRNAs that are targeted by small RNA species, e.g. miRNAs or small interfering RNAs (siRNAs). To determine if HIV-1 TAR miRNAs associated with any of the Argonaute proteins, we immunoprecipitated Flag-Ago protein from HEK 293 cultured cells stably transfected with the previously described constructs, U6-shNEG or U6-TAR [41] and overexpressing Flag-Ago 1, 2, 3 or 4 (Figure 1A). We analyzed immunoprecipitates by Northern blot using a radiolabeled probe against miR-TAR-3p and detected the mature miR-TAR-3p strand in all Flag-Ago immunoprecipitates (Figure 1B, probe miR-TAR-3p). The abundance of miR-TAR-3p in the precipitates correlated with the amounts of Ago protein expressed and immunoprecipitated (Figure 1A). We could not detected miR-TAR-5p in Northen Blot but we detected it in Ago1 and Ago2 immunoprecipitates when we used more sensitive RNAse protection assays (RPA) (Additional file 1, probe −5/32). These results suggest that TAR miRNAs could regulate host mRNA expression through their association with Argonaute effector complexes.

### A proteomic-based approach to identify cellular mRNA targets of HIV-1 TAR miRNAs

First, we used a proteomic-based approach to identify genes regulated by the HIV-1 TAR miRNAs. We created Jurkat cell lines that stably expressed either U6-shNEG or U6-TAR constructs and confirmed the expression of miR-TAR-5p and miR-TAR-3p (Additional file 2A) in four TAR-expressing Jurkat cell lines (Jurkat-TAR-1 to TAR-4) by RPA, using the −5/32 and 33/59 radiolabeled probes respectively [23] (Additional file 2B). Cell cycle regulation was assessed by flow cytometry and no difference was observed among Jurkat-TAR 1–4, the NEG-1 and wild-type (WT) Jurkat cell lines, suggesting that expression of HIV-1 TAR does not have cytotoxic effects on Jurkat cells (Additional file 3).

**Figure 2:**
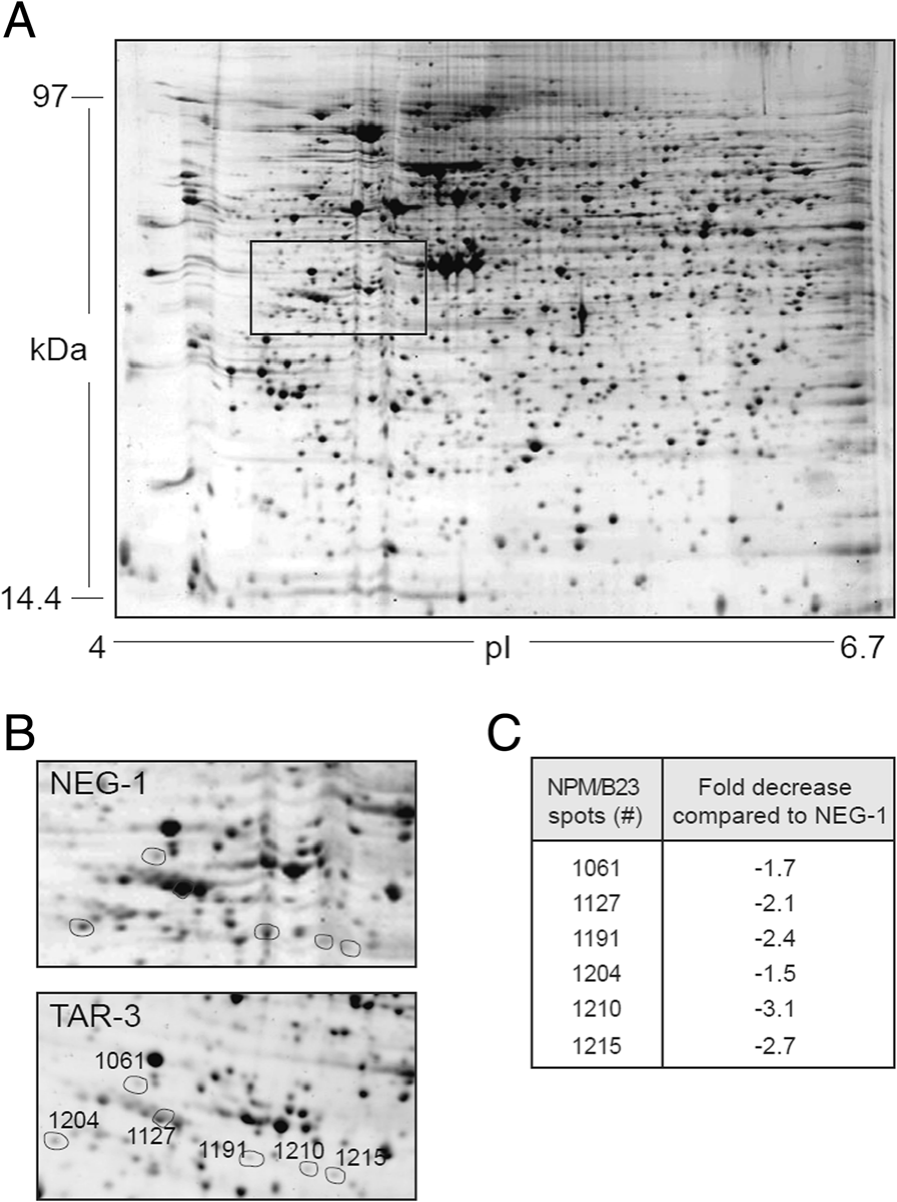
**NPM/B23 protein expression is downregulated in Jurkat cells stably expressing HIV-1 TAR. A)** Two-dimensional gel electrophoresis [isoelectric point (pI) range: 4 to 6.7] comparing clones of stable Jurkat cell lines expressing U6-TAR (TAR-3) or U6-shNEG (NEG-1). **B)** A region of the panel in A) is enlarged to show spots of the downregulated NPM/B23 protein in TAR-3 cell line (bottom left panel) compared to the control NEG-1 cell line (upper right panel). The six (6) spots identified as NPM/B23 (see results for details) are circled (TAR-3 panel). **C)** Proteomic analysis of the spots identified in B) showed there was a 1.5 to 3-fold decrease in the expression of the NPM/B23 protein in the Jurkat TAR-3 cell line, compared to Jurkat NEG-1 cells.

Of four clones tested, Jurkat-TAR-3 cells expressed the highest levels of TAR miRNA (Additional file 2B). When compared to Jurkat-NEG-1 control cells by 2D-gel electrophoresis and mass spectrometry, we found that NPM/B23, a 294-amino acid protein with a predicted isoelectric point of 4.78, was downregulated in TAR expressing cells (Figures 2A and 2B). A total of 6 spots representing many different protein species in the same area of the gel were identified as NPM/B23 (Figures 2A and 2B), and were downregulated close to three-fold in Jurkat-TAR-3 cells (Figure 2C). The different spots most likely represented forms of NPM/B23 proteins that had different post-translational modifications [42]. A similar 2D-gel electrophoretic analysis of the Jurkat-TAR-1 cell line, which expressed lower levels of TAR miRNAs (Additional file 2B), showed the expression of NPM/B23 protein was also downregulated, but to a lesser degree (D.L.O., J.J.R and P.P., unpublished observations), suggesting that TAR miRNAs regulate the expression of NPM/B23 in a dose-dependent manner.

To verify that the downregulation of NPM/B23 observed in the Jurkat-TAR cells was not specific to our Jurkat cell model, we also transiently transfected HEK 293 cells. Along with U6-TAR and U6-NEG plasmids, we used a psiSTRIKE-based vector expressing either miR-TAR-5p or miR-TAR-3p in the form of a short hairpin RNA (shRNA) (U6-sh5p and U6-sh3p respectively; Additional file 4A). These vectors were validated to individually express functional miR-TAR-5p and miR-TAR-3p using sequence-specific sensor constructs (Additional file 4B). Western blots revealed that NPM/B23 expression was also downregulated in HEK 293 cells that expressed TAR miRNAs (Additional file 4C), confirming our previous findings for NPM/B23 in Jurkat cells.

### A bioinformatic-based approach to identify cellular mRNA targets of HIV-1 TAR miRNAs

**Figure 3:**
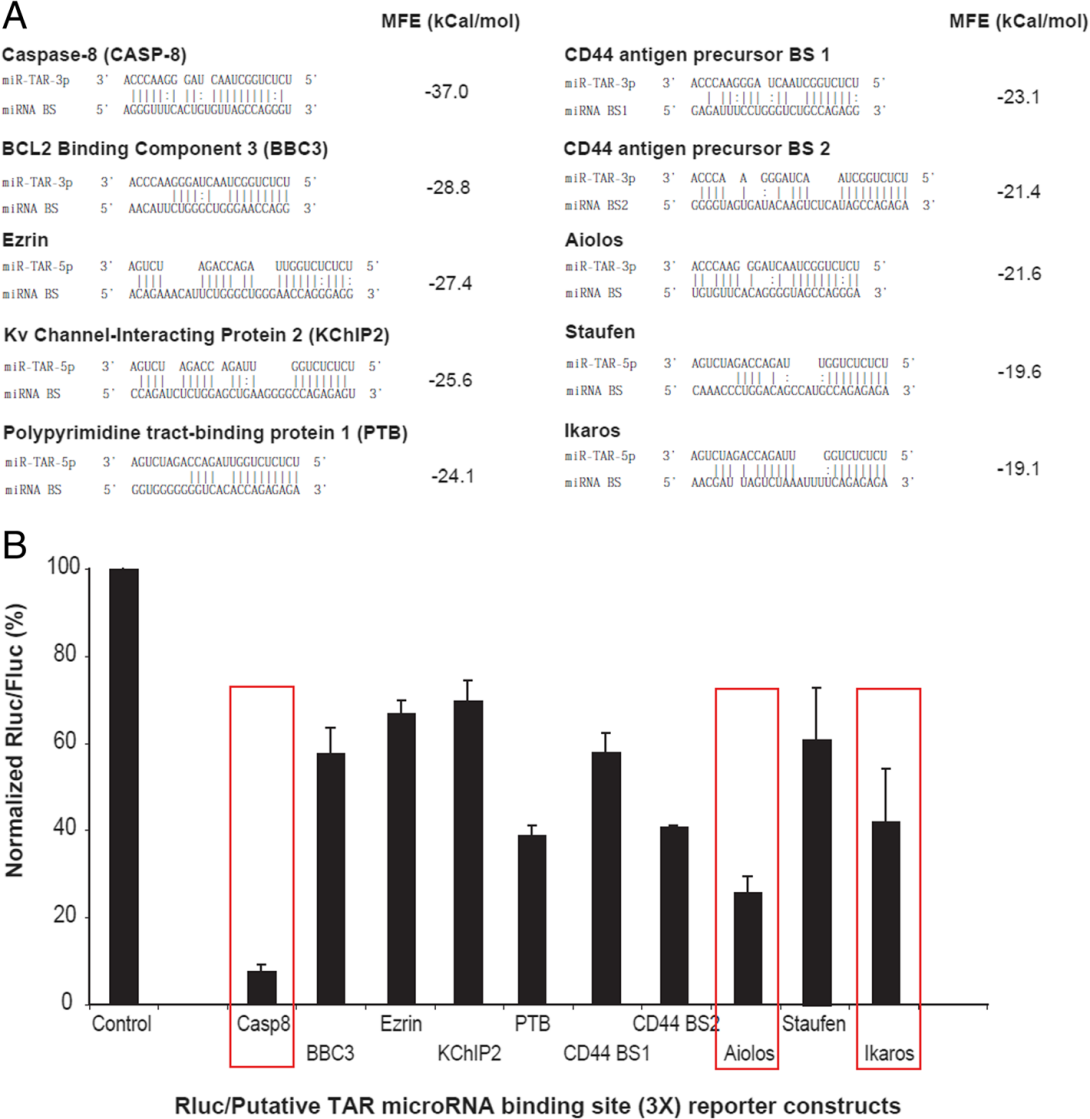
**HIV-1 TAR miR-TAR-5p and miR-TAR-3p target different subsets of host genes. A)** Minimal free energy (MFE) was calculated for a subset of mRNAs selected from the list produced by human miRTAR algorithm. **B)** HEK 293 cells were co-transfected with U6-shNEG (control) or U6-TAR and a vector containing the *Renilla* luciferase (Rluc) reporter gene usptream of three copies (3X) of the natural TAR-miRNA binding sites from the selected mRNAs (n = 1 to 3 experiments, in duplicate). Results are expressed as mean ± s.e.m.

We used an *in silico* approach, based on a previous version (2008) of the miRTAR algorithm (http://mirtar.mbc.nctu.edu.tw/), to search for human mRNAs that contain a putative binding site(s) in their 3’ UTRs for HIV-1 miR-TAR-5p and/or miR-TAR-3p. The algorithm generated a list of several candidate mRNAs with potential binding sites that could be recognized by miR-TAR-5p (Additional file 5) and/or miR-TAR-3p (Additional file 6). Minimal free energy (MFE) was determined by miRTAR or the RNA Hybrid algorithm (http://bibiserv.techfak.uni-bielefeld.de/rnahybrid/) and candidate mRNAs with different MFEs were selected and validated in reporter gene activity assays (Figures 3A and 3B). The ability of each TAR miRNA to downregulate mRNA containing putative miRNA binding sites from the candidate genes was established by inserting three copies of the candidate gene’s predicted miRNA binding sites into the 3’UTR of the *Renilla* luciferase (Rluc) gene and performing dual luciferase assays in HEK 293 cells for 48 hours (Figure 3B). Rluc expression was downregulated for all the candidate miRNA binding sites tested but no correlation could be established with the level of down regulation and the calculated MFEs (D.L.O., J.J.R and P.P., unpublished observations). The Caspase 8, Aiolos and Ikaros genes were selected for further investigations based on their level of regulation by TAR miRNAs, i.e.downregulation compare to control, and their relevance to HIV-1 pathogenesis.

### The binding sites for HIV-1 TAR miRNAs in the 3’ UTR of Caspase 8, Aiolos, Ikaros and NPM/B23 mRNAs are functional

**Figure 4:**
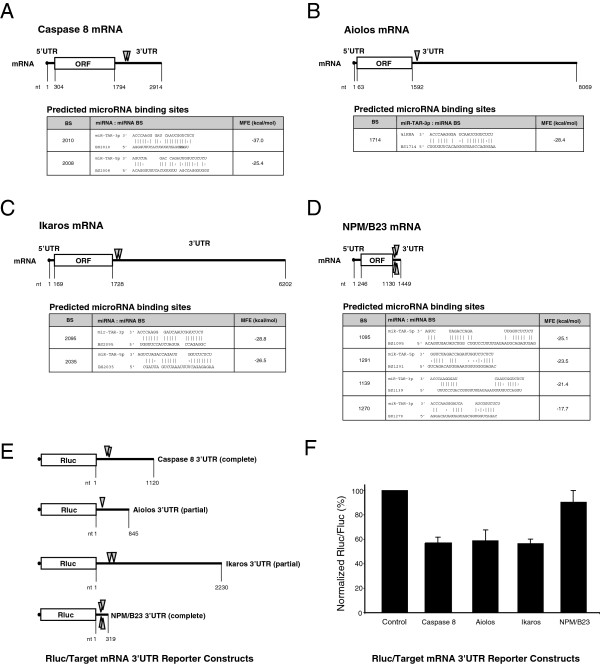
**The 3’ untranslated region of Caspase 8, Aiolos, Ikaros and NPM/B23 mRNAs are targeted by TAR miRNAs. A-D)** Schematic of the binding sites for HIV-1 miR-TAR-5p and/or miR-TAR-3p identified in the endogenous 3’UTR of Caspase 8, Aiolos, Ikaros and NPM/B23 mRNAs. Panels show the predicted base pairing and minimal free energy (MFE) calculated for each putative TAR miRNA binding site. **E)** Schematic of the constructs used for the dual luciferase assays, showing the *Renilla* luciferase (Rluc) reporter gene coupled to the complete or partial 3’ UTR of each mRNA. **F)** HEK 293 cells were co-transfected with U6-shNEG (control) or U6-TAR and and the reporter constructs described in panel E, (n = 1 to 3 experiments, in duplicate). Results are expressed as mean ± s.e.m.

The 3’UTR of Caspase 8, Aiolos, Ikaros and NPM/B23 mRNAs were analyzed by using the RNA Hybrid algorithm for the presence of binding sites to HIV-1 miR-TAR-5p and miR-TAR-3p (Figures 4A-D). The functionality of these binding sites was confirmed in HEK 293 cells by transiently transfecting them with U6-TAR and Rluc constructs that contained complete or truncated versions of the Caspase 8, Aiolos, Ikaros or NPM/B23 mRNA 3’UTRs (Figure 4E). For all constructs, Rluc expression was downregulated compared to control HEK 293 cells transiently transfected with the Rluc constructs and the U6-shNEG (Figure 4F). These results suggest a role for TAR miRNAs in regulating the expression of these genes through recognition of the miRNA binding elements located in their mRNA 3’UTRs.

The regulatory effects of TAR miRNAs on the reporter gene activity assays containing the NPM/B23 mRNA 3’UTR was relatively modest (Figure 4E, NPM/B23, ~10% downregulation). This may be understandable given the current biological understanding of miRNA function and the limitations of assays available for functional validation. A possible explanation is that putative miRNA binding sites within the 5’UTR or ORF of NPM/B23 mRNA could also contribute to the regulation of NPM/B23 expression *in vivo*. However, when tested, the 5’UTR and ORF of NPM/B23 mRNA did not downregulate the reporter gene activity in transiently transfected HEK 293 cells (Additional file 7). These data suggest that HIV-1 TAR miRNAs regulate NPM/B23 protein expression through the recognition of regulatory elements located mainly in the 3’UTR of NPM/B23 mRNA. The low effect might be explained by accessibility restrictions [43]for the TAR miRNAs on the NPM/B23 3’UTR fused to the *Renilla* luciferase gene. However, it appears that in mammals, viral miRNAs exert their regulatory effect using mostly the same mechanism as cellular miRNAs, by targeting the 3’UTR of mRNAs [44].

### Caspase 8, Aiolos, Ikaros and NPM/B23 mRNA levels are differentially regulated in TAR-expressing cell lines and J-Lat cell lines

**Figure 5:**
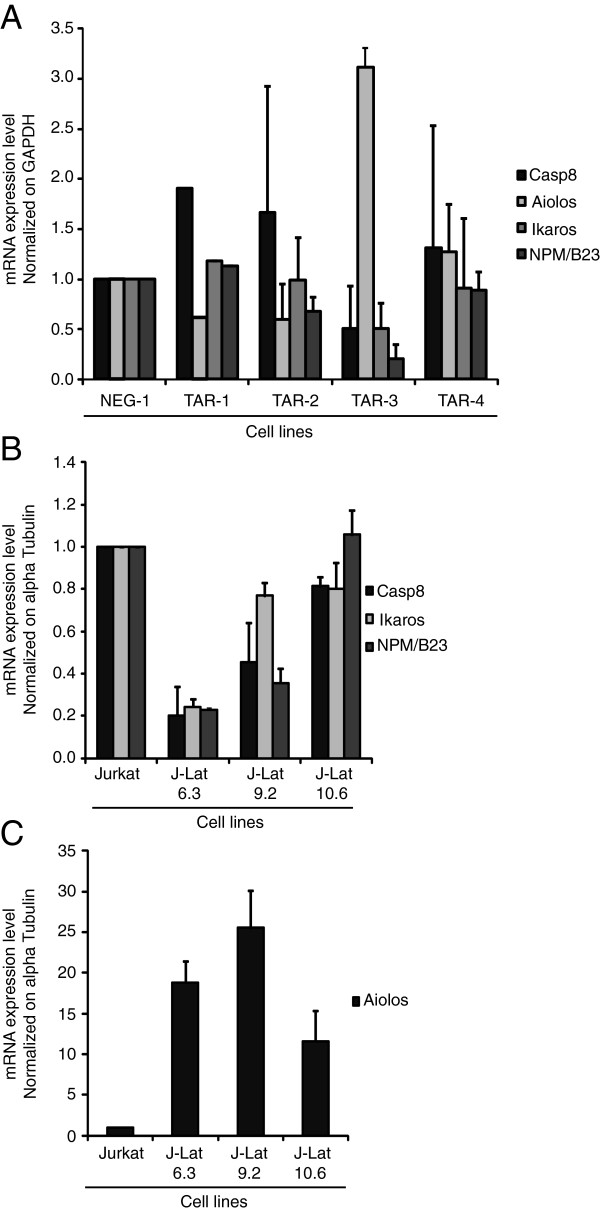
**Caspase 8, Aiolos, Ikaros and NPM/B23 mRNA levels are differentially regulated in Jurkat TAR cells and J-Lat clones. A)** mRNA expression of Caspase 8, Aiolos, Ikaros and NPM/B23 in Jurkat TAR clones 1–4 and NEG-1. Relative abundance was calculated by normalizing to GAPDH mRNA levels and the mRNA expression in the NEG-1 cells for each gene was set to 1. **B)** mRNA expression of Caspase 8, Ikaros and NPM/B23 in J-Lat clones 6.3, 9.2 and 10.6. Relative abundance was calculated by normalizing to α-Tubulin RNA levels and the expression in the WT Jurkat cell line was arbitrarily set to 1. **C)** mRNA expression of Aiolos in J-Lat cells. Relative abundance was calculated by normalizing to α-Tubulin RNA levels and the Aiolos expression in the WT Jurkat cell line was arbitrarily set to 1.

Cellular miRNAs normally exert their regulatory effects by repressing mRNA translation and/or mRNA degradation. Therefore, we examined if the Caspase 8, Aiolos, Ikaros and NPM/B23 mRNA levels were affected by the concomitant expression of TAR miRNAs in Jurkat cells. Quantitative RT-PCR analyses revealed that the Caspase 8, Ikaros and NPM/B23 mRNAs were less abundant in the TAR-3 Jurkat cell line compared to control Jurkat cells (Figure 5A), suggesting a destabilization of these mRNAs. In contrast, the levels of these mRNAs in the TAR-1, TAR-2 and TAR-4 Jurkat cell lines, which expressed lower levels of TAR miRNAs than TAR-3 (Additional file 2B), were similar to those observed in NEG-1 Jurkat cells (Figure 5A). This finding suggests that the amount of miRNAs produced from TAR, and incorporated into silencing complexes in the TAR-1, TAR-2 and TAR-4 Jurkat cell lines was sufficient to regulate expression of these genes through suppression of translation, without destabilizing the mRNA. On the other hand, Aiolos expression was regulated differently from the other genes in the Jurkat-TAR-3 cell line. Instead of being downregulated, Aiolos RNA was more abundant in the TAR-3 Jurkat cells than any other cell lines (Figure 5A). Whether or not this upregulation of Aiolos gene in TAR-3 correlate with the high level of TAR miRNAs expressed in this cell line (Additional file 2B) is unknown.

To determine if our observations in TAR-expressing cell lines were relevant to the whole virus, we examined the expression of Caspase 8, Aiolos, Ikaros and NPM/B23 mRNAs in J-Lat T cells, which are Jurkat cell lines that contain a full-length, latent HIV-1 provirus with an integrated green fluorescent protein gene (GFP) replacing the HIV-1 *nef* gene [45]. These cells are a well-known model used in reactivation of HIV-1 latency and they express short abortive TAR RNA transcripts [14] which are a substrate for Dicer and a source of TAR miRNAs [23,24]. J-Lat clones were selected based on a previously published study showing that unstimulated J-Lat clone 6.3 produces short transcripts containing TAR RNA in the amount of ~10 copies per cell [14]. We screened four J-Lat clones where the integrated proviruses reacted differently to the epigenetic-modifying agents histone deacetylase (HDAC) inhibitors and NF-kB/PKC activators (Additional file 8), and compared the Caspase 8, Aiolos, Ikaros and NPM/B23 expression to a WT Jurkat cell line.

The levels of the mRNAs encoding Caspase 8, Ikaros and NPM/B23 were lower in J-Lat clones 6.3 and 9.2 compared to WT Jurkat cells, but similar to WT in J-Lat clone 10.6 (Figure 5B). J-Lat clone 10.6 differs slightly from the other J-Lat clones; it is more susceptible to HDAC inhibitors (HDAC) and NF-kB/PKC activators, compared to the other J-Lat clones (Additional file 8), as demonstrated by a higher percentage of reactivation i.e. GFP levels (Additional file 8). Similar to the Jurkat-TAR-3 line, we detected the Aiolos mRNA at much higher levels in all the J-Lat clones compared to WT Jurkat cells (Figure 5C), albeit at a lower level in J-Lat clone 10.6. The fold increase increased by ~19%, ~25% and ~12% J-Lat clones 6.3, 9.2 and 10.6 respectively compared to WT Jurkat. As the data were collected in non-activated J-Lat cells, it is not clear how discrepancy between clones were linked to mRNA level analyses. At this point, it was not clear if HIV-1 TAR miRNAs were repressing the translation of Aiolos mRNA into protein.

### Caspase 8, Aiolos, Ikaros and NPM/B23 protein expression is altered in TAR-expressing cell lines and in J-Lat cell lines

Because miRNAs are known to bind mRNAs and cause an initial repression of mRNA translation [46], we analyzed the expression of the Caspase 8, Aiolos, Ikaros and NPM/B23 proteins in Jurkat-TAR cells and J-Lat cell lines by Western blotting. The regulation of these genes at the protein level may have an important impact at different stages of the HIV-1 pathogenic cycle.

**Figure 6:**
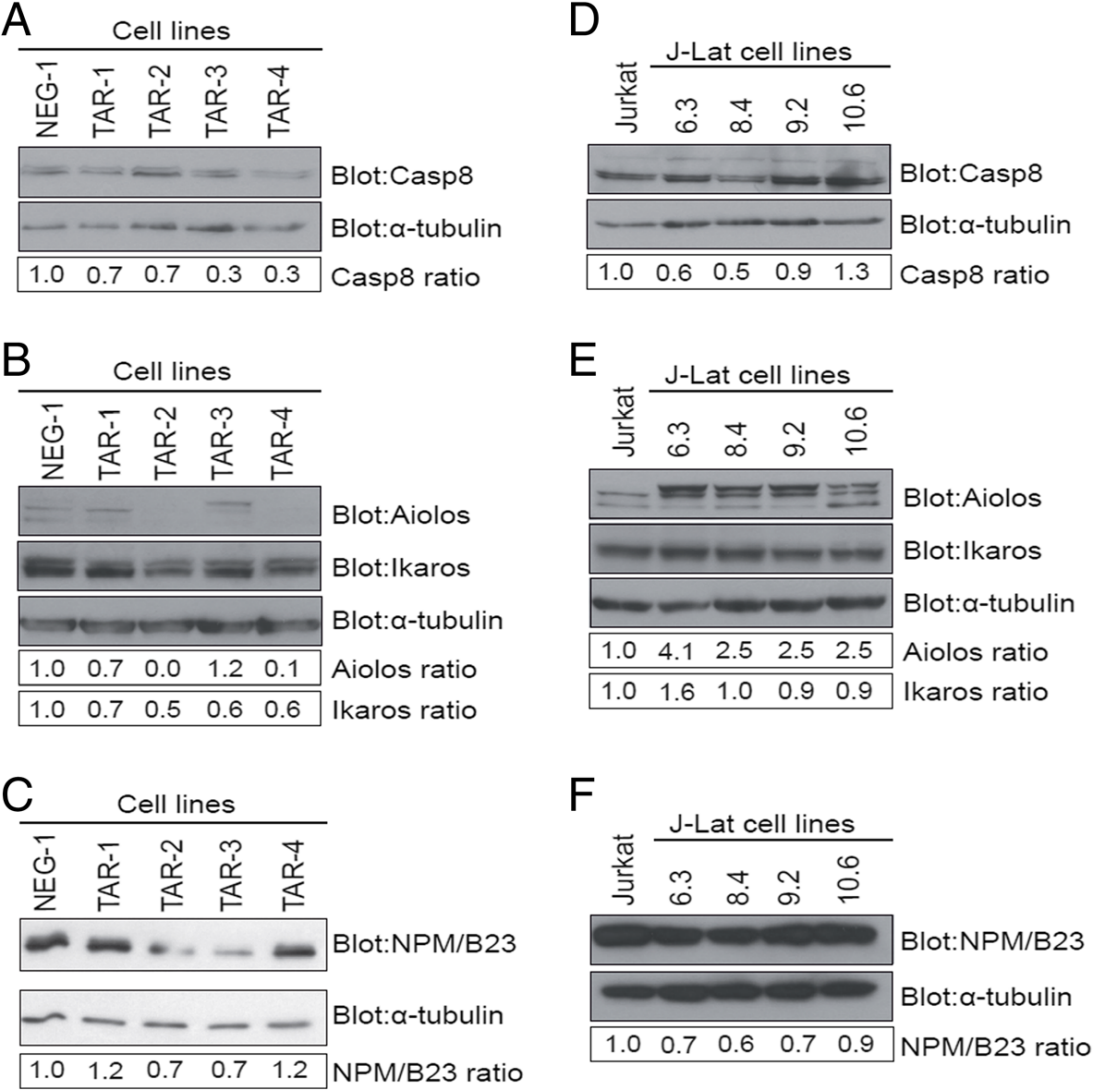
**Caspase 8, Aiolos, Ikaros and NPM/B23 protein expression is differentially regulated in Jurkat TAR-expressing and J-Lat clones.** Western blots of Caspase 8 **(A and D)**, Aiolos and Ikaros **(B and E)** and NPM/B23 **(C and F)** protein expression in Jurkat TAR cell lines (A-C) and J-Lat (C-F) cell lines. The relative abundance of the Caspase 8, Aiolos, Ikaros and NPM/B23 proteins were calculated by normalizing to the expression of α-tubulin protein in each sample. Samples were separated by 10% (B, E) or 15% SDS-PAGE (A, C, D and F).

Caspase 8 protein expression was downregulated between 30% and 70% in the Jurkat-TAR cells versus the negative control (Figure 6A). However, the extent of downregulation did not correlate with the level of TAR miRNA expression. For example, a 70% downregulation of Caspase 8 was observed in TAR-3 and TAR-4, which expressed the highest and the lowest amount of TAR miRNAs, respectively, according to the RPA performed on the Jurkat-TAR cell lines (Additional file 2B). The presence of two binding sites with highly favourable MFE values for miR-TAR-5p and miR-TAR-3p with their binding sites (−25.4 kcal/mol and −37.0 kcal/mol respectively; Figure 4A), suggested that Caspase 8 mRNA is sensitive to TAR miRNA regulation. However, the protein downregulation of Caspase 8 did not go above 70% and suggest that a basal level of Caspase 8 is required for cell survival and homeostasis. It is known that, in response to an apoptotic stimulus, the activated form of Caspase 8 protein is cleaved into smaller fragments. We did not observe any active Caspase 8 fragments on our immunoblots (D.L.O., J.J.R and P.P., unpublished observations), suggesting a stimulus-independent mechanism of downregulation.

Ikaros protein expression in Jurkat-TAR cell lines was downregulated from 30% to 50% (Figure 6B, Ikaros panels) depending on the cell line. As shown above for Caspase 8, Ikaros downregulation does not seem to be TAR mirna dose-dependent. NPM/B23 was also downregulated in the TAR-2 and TAR-3 cell lines by 30% (Figure 6C). Unlike Caspase 8 and Ikaros, NPM/B23 protein expression is lower in Jurkat-TAR cell lines expressing more TAR miRNAs (Figure 3C).

In contrast to the three other genes, but consistent with the mRNA data, we saw increased levels of Aiolos protein in the Jurkat-TAR cell lines (Figure 6B, Aiolos panels), so we cannot exclude the possibility that the TAR miRNAs were upregulating Aiolos gene expression. We observed a higher molecular weight Aiolos protein species in Jurkat-TAR-3 cells that was absent from the Jurkat-NEG-1 and other Jurkat-TAR cell lines (Figure 6B, Aiolos panels) and could be due to expression of the longest isoform of Aiolos, called Aio-1 [47].

To determine if the integrated HIV-1 provirus had an effect on the protein expression of the TAR-miRNA putative target genes, we monitored the levels of Caspase 8, Aiolos, Ikaros and NPM/B23 proteins in J-Lat cell lines. We saw a modest downregulation of Caspase 8 (~10% to 50%) and NPM/B23 (~10% to 40%) protein levels in J-Lat cells compared to WT Jurkat cells (Figure 6D and 6F). Although the Ikaros mRNA levels were downregulated by ~75% in J-Lat 6.3 and by ~20% in J-Lat 9.2 and 10.6 J-Lat, the protein level was only slightly reduced (~10% in J-Lat 9.2 and 10.6, nothing in other cell lines) (Figure 6E, Ikaros panels). In contrast, we observed an increase in Aiolos protein expression in all the J-Lat cell clones tested (Figure 6E, Aiolos panels), which correlated with the relatively high levels of Aiolos mRNA detected in these cells (Figure 6B, Aiolos panels).

In summary, the mRNA regulatory properties of TAR miRNAs were similar, at the protein level, in the Jurkat-TAR and J-Lat cell lines. In both models, we observed a downregulation of Caspase 8 and NPM/B23 protein levels, and an upregulation of Aiolos protein expression that was associated with HIV-1 TAR miRNA expression. Together, these observations suggest that a latent form of the HIV-1 virus generates TAR miRNAs that could regulate expression of genes, at the protein level, and could potentially be important for cell survival and affect HIV-1 replication.

### The ratios between proteins regulated by HIV-1 miRNAs may play a role in apoptosis resistance of HIV-1 infected cells

**Figure 7:**
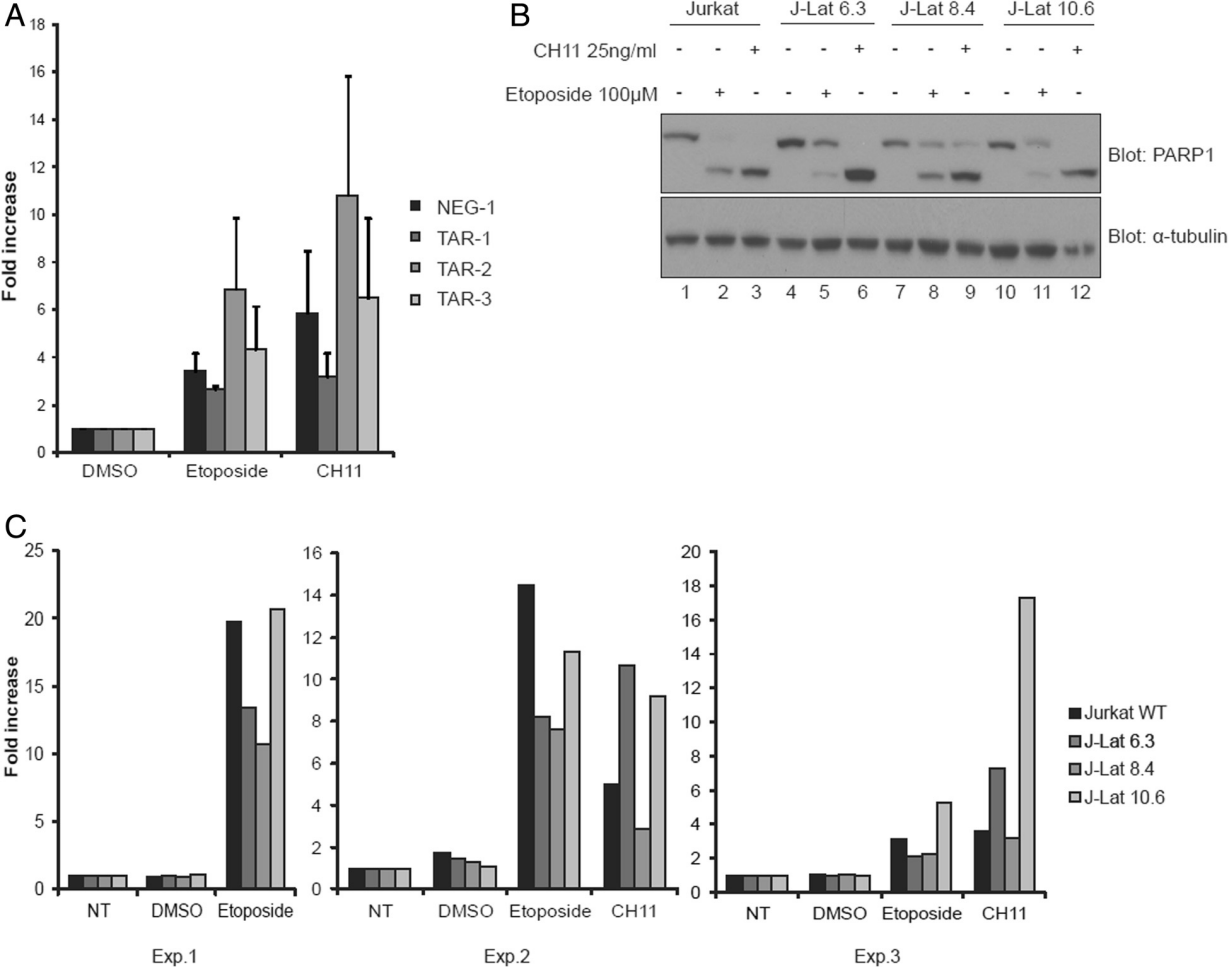
**Expression of TAR and TAR miRNAs may not protect Jurkat cells from apoptosis. A)** Apoptosis of Jurkat NEG-1 and Jurkat-TAR cell clones 1, 2 and 3 was induced by treating with etoposide (100 μM) or CH11 anti-fas activating antibody (25 ng/ml) for 6 hours. Cells were analyzed using a luminescent assay for Caspase 3 and 7 activity. Data are presented as fold increases of relative luminescent units compared to cells treated with 1% DMSO. B and C) Apoptosis of J-Lat cell clones 6.3, 8.4 and 10.6 was induced by treatment with etoposide (100 μM) or CH11 anti-fas activating antibody (25 ng/ml) for 6 hours. **B)** Cells were lysed and proteins were separated on a 10% polyacrylamide gel and analyzed by Western blot using antibodies against PARP-1 and α-tubulin. **C)** For Caspase 3 and 7 activities, the assay was performed according to the manufacturer’s instructions and luminescence detection was assessed. The results of three (3) independent experiments are shown separately.

Apoptosis is an important hallmark of HIV-1 pathogenesis [33] and it is not clear how HIV-1 interacts with the cellular machinery to promote cell survival or avoid apoptosis. To examine the impact of the TAR miRNA regulation of Caspase 8, Aiolos, Ikaros and NPM/B23 protein levels on apoptosis, we induced apoptosis in both the Jurkat-TAR cells (Figure 7A) and the J-Lat cell clones (Figure 7B and 7C) by treating with etoposide and anti-fas CH11. In the cell, etoposide complexes with topoisomerase II and DNA to cause double and single-stranded DNA breaks that induce apoptosis through the DNA repair pathway. Anti-fas CH11 is an antibody that activates the CD95/Fas receptor and induces apoptosis by stimulating the Fas receptor, and subsequently activating the caspases. Resistance to one or other drug might allow us to link the genes targeted by the TAR miRNAs to the DNA damage response pathway or the Fas receptor pathway.

Apoptosis was monitored by measuring the enzymatic activity of the effector proteins Caspase 3 and 7, both active with etoposide and CH11 treatments. We observed either no difference (Figure 7A, TAR-1 and TAR-3) or a slightly higher rate of apoptosis (Figure 7A, TAR-2) in Jurkat TAR cell lines compared to the control cells NEG-1, which express a shRNA under U6 promoter transcription (U6-shNeg). Therefore, the downregulation of NPM/B23 protein levels and upregulation of Aiolos protein expressed in the Jurkat-TAR-3 cell line may not affect the balance of the cells between survival and apoptosis. Both the etoposide and CH11 apoptotic agents induced apoptosis in WT Jurkat and J-Lat cells, confirmed by the detection of cleaved Poly [ADP-ribose] polymerase 1 (PARP-1) (Figure 7B), a downstream target of Caspase 3, on immunoblots [48]. However, all of the J-Lat clones had reduced amounts of cleaved PARP-1 compared to WT Jurkat cells (Figure 7B, clones 6.3, 8.4 and 10.6), indicating a certain degree of resistance to apoptosis. Furthermore, J-Lat clones were more resistant to apoptosis induced by DNA breaks than by CD95/Fas activation as we observed almost complete cleavage of PARP-1 in J-Lat clones 6.3, 8.4 and 10.6 treated with CH11 (Figure 7B, lane 6, 9 and 12).

The resistance of J-Lat cells to apoptosis was also analyzed using a Caspase 3/7 assay. After treating with etoposide, J-Lat cell clones 6.3 and 8.4, but not 10.6, had reduced caspase activity compared to treated WT Jurkat (Figure 7C, etoposide, all panels). However, the J-Lat cell lines, except for J-Lat clone 8.4, were more susceptible than WT Jurkat cells to apoptosis when it was induced by the anti-fas CH11 antibody. This finding is consistent with the reduced amount of cleaved PARP-1 shown in Figure 7B for the same clones.

Our results indicate that HIV-1 infected cells are more resistant to apoptosis when it is induced by the DNA damaging agent etoposide. These data suggest that when an HIV-1 genome is integrated into the host DNA, several host genes including Caspase 8, Ikaros, Aiolos and NPM/B23 genes are regulated by the HIV-1 TAR miRNAs. The downregulation Caspase 8, Ikaros and NPM/B23 of, and the overexpression of Aiolos, may play important roles in the cell fate after HIV-1 infection.

## Discussion

HIV-1 regulates the expression of many host cell genes during infection of human cells [49]. Dysregulation of host genes occurs when viral molecules interact with cell components, disrupt normal cellular pathways, recruit host factors for viral replication or change the endogenous miRNA profile of the cell [50-53]. Here, we report that HIV-1 TAR miRNAs could be another mechanism by which HIV-1 regulates the expression of host genes. We demonstrated that miR-TAR-5p and miR-TAR-3p were incorporated into Ago complexes, which contains the effector components of miRNA-targeted RNA silencing [23] in human cells [54] and is required for small RNA species to mediate mRNA regulatory effects [5]. We established stable Jurkat cell lines expressing the HIV-1 TAR miRNAs and we selected four lines that showed variations in TAR miRNA expression levels. Based on the premises that (i) miRNAs regulate mRNAs in a dose-dependent manner [55], and that (ii) increased miRNA levels would yield a stronger phenotype, the Jurkat TAR-3 cell line was selected for further proteomic analyses and NPM/B23 emerged as a top protein candidate downregulated by the HIV TAR miRNAs. The 2D-gel analysis of a clonal population (i) circumvented a relatively high background associated to poorly infected cells, as previously described [56], and (ii) allowed us to study a stable latently-infected cell population, in contrast with other early-infected cell studies [56,57]. This approach, however, also has its intrinsic limitations, the most important of which may be (i) the poor separation of proteins from some areas of the gel, and (ii) a fold change too small to justify mass spectrometry analyses.

A dual luciferase reporter assay was used to confirm the host gene targets we identified *in silico* and to monitor the 3’UTR-mediated regulation of the genes of interest by the TAR miRNAs. The dual luciferase assay, although powerful, has limitations, all of which can differently influence the results [58]. These include the type of cells studied, the length of the regulatory element (e.g. natural 3’UTR versus 3X binding sites), the binding site accessibility and the architecture of the miRNA:mRNA base pairing. Data should be interpreted with caution, because modest reporter gene downregulation (~20%) has been previously observed in assays where the 3’UTR tested was fused to luciferase genes [31,59], even when the predicted binding sites had a free energy (ΔG) as low as −30,7 kcal/mol, expecting a much stronger regulation [31].

Three of the four genes that we selected from our reporter gene activity data were downregulated by TAR miRNAs expressed in the Jurkat cell model. Although the protein levels of Caspase 8, Ikaros and NPM/B23 were downregulated to varying degrees in the Jurkat cells expressing TAR and the J-Lat cell lines, TAR miRNAs did not induce comparable changes at the mRNA level. Therefore, although reduced mRNA levels generally account for most of the decreased protein production [60], it would be imprudent to rely exclusively on mRNA levels when assessing the effects and importance of TAR miRNAs in regulating host gene expression. Hence, the relevance of our proteomic analysis of Jurkat cells expressing TAR miRNAs that identified NPM/B23 as an mRNA/protein that is targeted by the HIV-1 TAR miRNAs.

The Aiolos gene was upregulated when TAR was expressed at a high level in the Jurkat cells or when the HIV-1 was integrated in the cells as a provirus. The upregulation of a gene upon miRNA binding has been observed with small activating RNAs (saRNAs), which are reported to enhance gene expression [61,62]. In addition, a given miRNA binding site can mediate repression in some 3’ UTRs, but not others [46], which could also explain the result. The overall effect of the TAR miRNAs on Aiolos expression may also involve regulation at the transcriptional level and, because Aiolos is a transcription factor, there may be complex feedback loops involved. The TAR miRNA downregulatory effect on Aiolos might be related to other stages of the HIV-1 pathogenesis or could occur in different cells types that have not been tested in this study.

Apoptosis is known to be a significant cause of HIV-1-infected, CD4^+^ T cell death. However, the molecular mechanism determining the balance between cell survival and cell death remains unclear. Our results suggest that the regulation of selected host mRNAs by TAR miRNAs could influence the choice made by HIV-1 infected cells between survival and apoptosis. This could be the result of a complex interplay involving the up- and down regulation of the levels of pro- and anti-apoptotic proteins by the TAR miRNAs, and the secondary effects on the cellular machinery. Furthermore, because HIV-1 infection is a multi-step process, these cell components may be regulated differently throughout the course of the infection. For instance, up-regulation of the pro-apoptotic Caspase 8, which cleaves and activates other downstream caspases, renders cells susceptible to apoptosis via Fas signaling [63]. Caspase 8 is cleaved by the HIV-1 protease into Casp8p41, a fragment strongly associated with apoptotic, HIV-1 infected CD4^+^ T cells [64]. HIV-1 could, for example, delay or prevent apoptosis through TAR miRNA-mediated downregulation of Caspase 8 early in the infection cycle, to ensure robust viral replication and packaging.

Another downregulated gene was Ikaros (also called IKZF1), which is the founding member of a family of zinc finger transcription factors that also includes Aiolos (also called IKZF3) [39]. Ikaros increases normal oxidative stress-induced apoptosis in erythroid cells [65]. By reducing the level of Ikaros protein in T cells, TAR miRNAs could decrease apoptotic events that are associated with HIV-1 infection. However, because etoposide treatment can shorten the half-life of Ikaros [66], we cannot exclude the effect of etoposide on Ikaros as an explanation for the resistance of the etoposide-treated Jurkat TAR and J-Lat cells to apoptosis.

Upregulation of Aiolos expression could also potentially reduce apoptosis of HIV-1 infected cells. Aiolos is a transcription factor whose expression is restricted to lymphoid lineages. Aiolos binds to the Bcl-2 promoter, and also interacts with the Bcl-2 and Bcl-X_L_ proteins [67,68] to enhance their stability and promote cell survival. Because a high Aiolos mRNA and protein expressions seemed to correlate with TAR miRNA levels and a resistance to apoptosis, it may be related to survival in infected cells.

The effects we have seen, e.g. cell survival and resistance to apoptosis, in TAR-expressing and J-Lat cells lines may also be explained by the downregulation of NPM/B23 protein. NPM/B23 is a major nucleolar, multifunctional protein that has been reported to interact with the HIV-1 proteins Tat and Rev [69,70] and with many cellular components [40]. A down regulation of NPM/B23 protein expression in the nucleolus could modify protein interactions and disrupt HIV-1 nucleolar Rev localization [71] to promote its interaction with other cellular or viral components. In the acute phase of HIV-1 infection, NPM/B23 expression is increased [69] and its acetylated form is recruited, in a Tat-dependant manner, to the HIV-1 LTR to enhance viral transactivation [72]. The targeting of NPM/B23 could be a mechanism used by the TAR miRNAs to limit the replication of HIV-1 thereby facilitating the escape of virus-infected cells from apoptosis and promoting latency stage.

The present study should improve our understanding of the regulation of host genes by viral miRNAs and the potential outcomes. Because hundreds of mRNAs can be targeted by a single miRNA, and miRNAs may act in concert to regulate mRNA translation [46], we speculate that the TAR miRNAs of HIV-1 have evolved to play a key role in viral pathogenesis, most likely by promoting conditions that favor HIV-1 replication in host cells. Future studies will elucidate the molecular mechanisms by which the HIV-1 miRNAs are produced and how they regulate host gene expression, to determine their relative importance in the HIV-1 replicative cycle and pathogenesis. These studies should provide key insights into how HIV-1 viral miRNAs contribute to shaping the host response to viral infection and identify new, potential targets for development of novel and improved therapeutics treat HIV-1.

## Conclusions

HIV-1 TAR miRNAs may regulate cellular apoptosis through the direct or indirect regulation of apoptosis-related genes, including Caspase 8, Aiolos, Ikaros and NPM/B23. The TAR miRNAs act through components of the regulatory pathway for host miRNAs, including the Ago complexes. HIV-1 miR-TAR-5p and miR-TAR-3p may thus determine the success of viral replication by regulating the balance between the survival and death of HIV-1 infected cells.

## Methods

### DNA constructs

The psiSTRIKE vector (Promega) encoding a short hairpin against a deleted region of *Firefly* luciferase gene was used as a negative control (U6-shNEG). The WT TAR region (U6-TAR) and psiCHECK (Promega) reporter construct encoding miR-TAR-5p (miR-TAR-5p sensor) and miR-TAR-3p (miR-TAR-3p sensor) have been described previously [23,41]. Vectors expressing short hairpin RNAs, encoding either miR-TAR-5p (U6-sh5p), or miR-TAR-3p (U6-sh3p), were cloned using sh5p up and down or sh3p up and down oligonucleotides into psiSTRIKE vector.

Three copies (3X) of the individual, natural binding sites of each selected mRNA target identified by bioinformatic analysis were inserted downstream of the Rluc open reading frame (ORF) into the XhoI/NotI restriction sites of a psiCHECK vector. The complete or truncated 3’UTR region of Caspase 8 [ENSG00000064012, complete 1120 base pair (bp) cloned], Aiolos [ENSG00000161405, first 845 bp cloned], Ikaros [ENSG00000185811, first 2230 bp cloned] and NPM/B23 mRNAs [ENSG00000181163, 319 bp cloned], as well as the 5’UTR and ORF of NPM/B23 mRNA, were amplified by the polymerase chain reaction (PCR), and then cloned into the XhoI/NotI restriction sites of psiCHECK vector. Oligonucleotide sequences are listed in Additional file 9.

### Mammalian cell culture

Jurkat and J-Lat (full length clones 6.3, 8.4, 9.2 and 10.6) were obtained from Dr. Eric Verdin through the NIH AIDS Research and Reference Reagent Program, Division of AIDS, NIAID. Cells were maintained in RPMI-1640 medium supplemented with 10% fetal bovine serum and 2 mM L-glutamine at 37°C in a humidified incubator under 5% CO_2_. HEK 293 and HEK 293 Flag-Ago 1, 2, 3, 4 cells [73] were grown in DMEM supplemented with 10% fetal bovine serum, 1 mM sodium pyruvate, 2 mM L-glutamine at 37°C in a humidified incubator under 5% CO_2_. Jurkat NEG-1 and TAR stable cell lines were established by transfection with either U6-shNEG or U6-TAR linearized vectors respectively, by electroporation and subsequent clonal selection using selective agent G418. Positive clones were selected for the presence and abundance of HIV-1 TAR miRNAs by using RNase protection assays (RPA) with probes and by methods previously described [23].

### Western blot

Cells were harvested and lysed in RIPA buffer (50 mM Tris–HCl pH 7.4, 150 mM NaCl, 1% NP-40, 0.1% SDS, 0.5% Sodium Deoxycholate,1 mM EDTA, 1X protease inhibitor cocktail) for 15 minutes on ice, and then centrifuged for 10 min at 10,000 g (4°C). Ten (10)-70 μg of protein were separated by 10-15% SDS-PAGE and transferred to PVDF membranes. Membranes were blocked in 5% non-fat milk then probed with primary antibodies for NPM/B23 (sc-47725, Santa Cruz Biotechnology), Caspase 8 (9746, Cell Signaling), Aiolos (sc-101982, Santa Cruz Biotechnology), Ikaros (sc-13039, Santa Cruz Biotechnology), Flag (F-3165, Sigma), α-tubulin (T6074, Sigma), GAPDH (2118, Cell signalling) overnight at 4°C. Anti-mouse or anti-rabbit were used as secondary antibodies and (Santa Cruz Biotechnology) and chemiluminescent signal was revealed using ECL substrate (Pierce).

### Immunoprecipitation

HEK 293 and HEK 293 Flag-Ago1, 2, 3 or 4 cell lines, were grown to 60% confluency, then transfected with U6-shNeg or U6-TAR. Forty-eight (48) hours later, cell were lysed in IP buffer (50 mM Tris–HCl pH 8,0, 137 mM NaCl, 1% Triton X-100, 1X protease inhibitor cocktail), and 3 mg of total lysate was incubated with anti-Flag M2 affinity agarose gel beads (A2220, Sigma) overnight in the presence of RNAse inhibitors. Beads were washed three times for five min in IP buffer and prior to the last wash, were divided as follows: one quarter (¼) of the IP was resuspended in 20 μl of loading dye containing β-mercaptoethanol and kept for Western blot analysis and three quarters (¾) was resuspended in 20 μl of nuclease-free water and kept for Northern Blot analysis. All samples were boiled for 10 minutes after resuspension, and the input (total lysate) was kept for protein analysis and total RNA extraction.

### Northern blot

Total RNA from inputs was extracted using Stat-60 reagent (Tel-Test, Inc) and RNA was collected from immunoprecipitates as described above, then separated by denaturating 8% PAGE. RNA was transferred onto Hybond-XL membrane (GE), UV crosslinked and hybridized in with PerfecHyb Plus (Sigma) containing radiolabeled [α-^32^P] ATP probes complementary to NEG RNA, miR-TAR-3p and U6 RNA (See Additional file 9 for the oligonucleotide sequences).

### Two-dimensional (2D) gel electrophoresis and proteomic analysis

Briefly, proteins were extracted from four different cultures of Jurkat NEG clone 1 and Jurkat TAR clone 3 in 7 M urea, 2 M thiourea, 3% 3-[(3-Cholamidopropyl) dimethylammonio]-1-propanesulfonate (CHAPS), 20 mM 1,4-Dimercapto-2,3-butanediol, 5 mM Tris(2-carboxyethyl) phosphine hydrochloride (TCEP) and precipitated using the 2D clean-up kit (GE Healthcare). Two hundred (200) μg of proteins was loaded onto a 24 cm Immobiline Dry Strip (GE Healthcare) for the first gel dimension. The second dimension was run on a 12% polyacrylamide gel and gels were fixed overnight then stained with Sypro Ruby (Invitrogen) for 5 h. Comparative analysis of the Jurkat NEG-1 and Jurkat TAR-3 was performed using the Progenesis software (Nonlinear Dynamics). Spots of interest were excised from the gels and trypsinized prior mass to spectrometry analysis. Peptide samples were separated by online reverse-phase (RP) nanoscale capillary liquid chromatography (nanoLC) and analyzed by electrospray mass spectrometry (ES MS/MS). Mass spectra were acquired using a data-dependent acquisition mode using the Xcalibur software (version 2.0). All MS/MS samples were analyzed using Mascot (version 2.2.0; Matrix Science). Scaffold (version Scaffold-2_01; Proteome Software Inc.) was used to validate MS/MS-based peptide and protein identification. Details are available upon request.

### Dual luciferase reporter gene activity assay

HEK 293 cells were co-transfected with U6-TAR vector (50 ng; Promega) and a psiCHECK reporter construct (500 ng) in which the Rluc reporter gene was upstream of three copies (3X) of the natural binding site of selected host mRNAs (n = 1 to 3 experiments, in duplicate). All Rluc/Fluc ratios were normalized with ratios from HEK 293 cells containing a psiNEG U6-shNEG control construct.

Using Lipofectamine 2000 (Invitrogen), HEK 293 cells were also co-transfected with U6-shNEG, U6-TAR, U6-sh5p or U6-sh3p (100–500 ng) and either the miR-TAR-5p or miR-TAR-3p sensors, or a psiC-NPM/B23 5’UTR, ORF or 3’UTR (0.5-50 ng per construct). Cells were harvested 24–48 hrs later and luciferase activities were measured as previously described [23,74]. Results are expressed as mean ± standard error of the mean (s.e.m.).

### Reverse transcription and quantitative PCR analysis

RNA was treated with Turbo™ DNAse (Life Technologies) for 30 minutes at 37°C and reverse transcribed (RT) with random primers and the M-MLV reverse transcriptase enzyme (Invitrogen), according to the manufacturer’s instructions. Quantitative PCR (qPCR) using IQ™ SYBR® green supermix (Bio-Rad) and listed primers sets (Additional file 9) was performed in a C1000 thermal cycler with a CFX96 Real-Time system (Bio-Rad). Data were collected using the Bio-Rad CFX manager software v. 1.6 (Bio-Rad).

### Induction of apoptosis

Jurkat WT and J-Lat cells were seeded, in triplicate, into 96-well plates and incubated in the presence of 1% v/v DMSO, 25 ng/ml Human Anti-Fas activating antibody (clone CH11, Millipore) or 100 nM Etoposide (Sigma) for 6 hours. Caspase-3 and Caspase-7 activities were detected using the Caspase-Glo 3/7 assay (Promega) according to the manufacturer’s recommendations, or the proteins were extracted from the cells and analyzed by Western blotting using anti-PARP antibody.

## Competing interest

The authors declare that they have no competing interest.

## Authors’ contributions

DLO, JJR and PP conceived the study; DLO, JV-E, KL, L-AG, IP and JCB performed the experiments, DLO, JV-E and PP analyzed the data; DLO and PP wrote the manuscript. All authors read and approved the final manuscript.

## Supplementary Material

Additional file 1 HIV-1 TAR miRNA are loaded into Argonaute 1 and 2 complexes. RNase protection assay analyses of RNA extracted from anti-Flag immunoprecipitates derived from HEK 293 cells expressing Flag-Argonaute 1 (Flag-Ago1) or Flag-Argonaute 2 (Flag-Ago2). Click here for file

Additional file 2 Expression of TAR-derived miR-TAR-5p and miR-TAR-3p in four (4) different TAR-expressing Jurkat cell lines. Consensus miR-TAR-5p:miR-TAR-3p sequences from HIV-1 TAR TAR RNA and RNase protection assays using probes against miR-TAR-5p and miR-TAR-3p for RNA extracted from Jurkat TAR-expressing cell lines (clones TAR 1, 2, 3 and 4) and the control (NEG-1). Click here for file

Additional file 3 Cell cycle analysis for Jurkat TAR-expressing and NEG cell lines. Analysis of the cell cycle by flow cytometry in Jurkat TAR-expressing cell lines (Jurkat TAR 1, 2, 3 and 4) and in control cells (Jurkat WT and NEG-1) using propidium iodide (PI). Click here for file

Additional file 4 NPM/B23 protein expression is downregulated by both miR-TAR-5p and miR-TAR-3p. Schematic, reporter assay and Western blots showing U6-sh5p and U6-sh3p downregulated NPM/B23 in HEK 293 cells. Click here for file

Additional file 5 miRTAR predictions for HIV-1 miR-TAR-5p targets. List of the messenger RNAs targeted by miR-TAR-5p, based on miRTAR predictions. Click here for file

Additional file 6 miRTAR predictions for HIV-1 miR-TAR-3p targets. List of the messenger RNAs targeted by miR-TAR-3p, based on miRTAR predictions. Click here for file

Additional file 7 Both miR-TAR-5p and miR-TAR-3p regulate gene expression through the 3’UTR of NPM/B23. Reporter gene assay showing the regulation of NPM/B23 5’UTR, ORF and 3’UTR by the miR-TAR-5p or miR-TAR-3p, when expressed individually from conventional stem-loop. Click here for file

Additional file 8 Drug stimulation of J-Lat cell lines. Flow cytometry analysis of J-Lat clones 6.3, 8.4, 9.2 and 10.6 stimulated with TNF-α, PMA, prostatin, TSA, SAHA, valproic acid and HMBA for 18 hours. Click here for file

Additional file 9 Oligonucleotide sequences. List of oligonucleotide sequences used for cloning, PCR and as DNA probes. Click here for file
