# Supplementary material for: Regulation of host gene expression by HIV-1 TAR microRNAs
Source: Retrovirology. 2013 Aug 12;10:86. doi: 10.1186/1742-4690-10-86 (PMC3751525; doi:10.1186/1742-4690-10-86)
Supplement: Additional file 1 — HIV-1 TAR miRNA are loaded into Argonaute 1 and 2 complexes. RNase protection assay analyses of RNA extracted from anti-Flag immunoprecipitates derived from HEK 293 cells expressing Flag-Argonaute 1 (Flag-Ago1) or Flag-Argonaute 2 (Flag-Ago2). [file 1742-4690-10-86-S1.pdf]

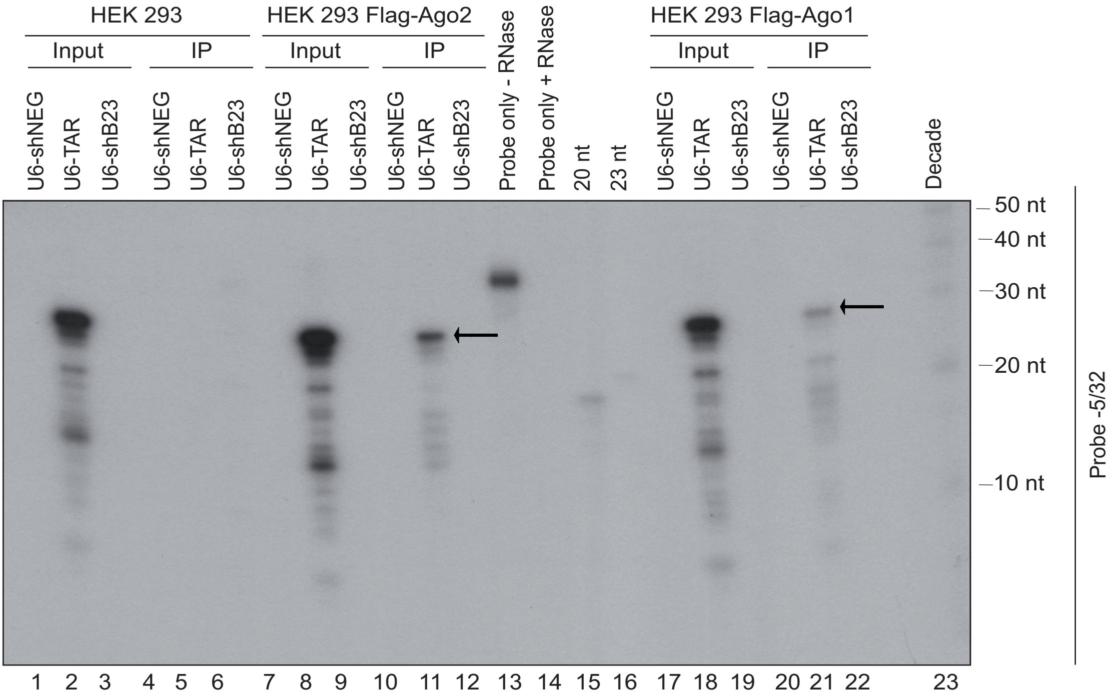

Additional File 1. HIV-1TAR miRNA are loaded into Argonaute 1 and 2 complexes. HEK293, HEK 293 Flag-Ago1, HEK 293 Flag-Ago2 cell lines were transfected with U6-sh-NEG, U6-TAR or U6-shB23 (use as a negative control). Forty-eight (48) hours later, cell were lysed in IP buffer (50 mM Tris-HCl ph 8,0, 137 mM NaCl, 1% Triton X-100, 1X protease inhibitor cocktail) and 3 mg of total lysate was incubated with anti-Flag M2 affinity agarose gel beads (Sigma) overnight at 4°C on a rotator. Beads were washed 3 x 5 minutes in IP buffer and before the last wash, beads were divided. A quarter of the IP was resuspended in 20 µl of loading dye and kept for western blot analysis, three quarter of the IP was resuspended in 20 µl of nuclease-free water and kept for RNase protection assay (RPA). All samples were boiled for 10 minutes after resuspension and input (total lysate) was kept for protein analysis and total RNA extraction using Stat-60. RNAse protection assay was performed as previously described [23].
