# Supplementary material for: Regulation of host gene expression by HIV-1 TAR microRNAs
Source: Retrovirology. 2013 Aug 12;10:86. doi: 10.1186/1742-4690-10-86 (PMC3751525; doi:10.1186/1742-4690-10-86)
Supplement: Additional file 2 — Expression of TAR-derived miR-TAR-5p and miR-TAR-3p in four (4) different TAR-expressing Jurkat cell lines. Consensus miR-TAR-5p:miR-TAR-3p sequences from HIV-1 TAR TAR RNA and RNase protection assays using probes against miR-TAR-5p and miR-TAR-3p for RNA extracted from Jurkat TAR-expressing cell lines (clones TAR 1, 2, 3 and 4) and the control (NEG-1). [file 1742-4690-10-86-S2.pdf]

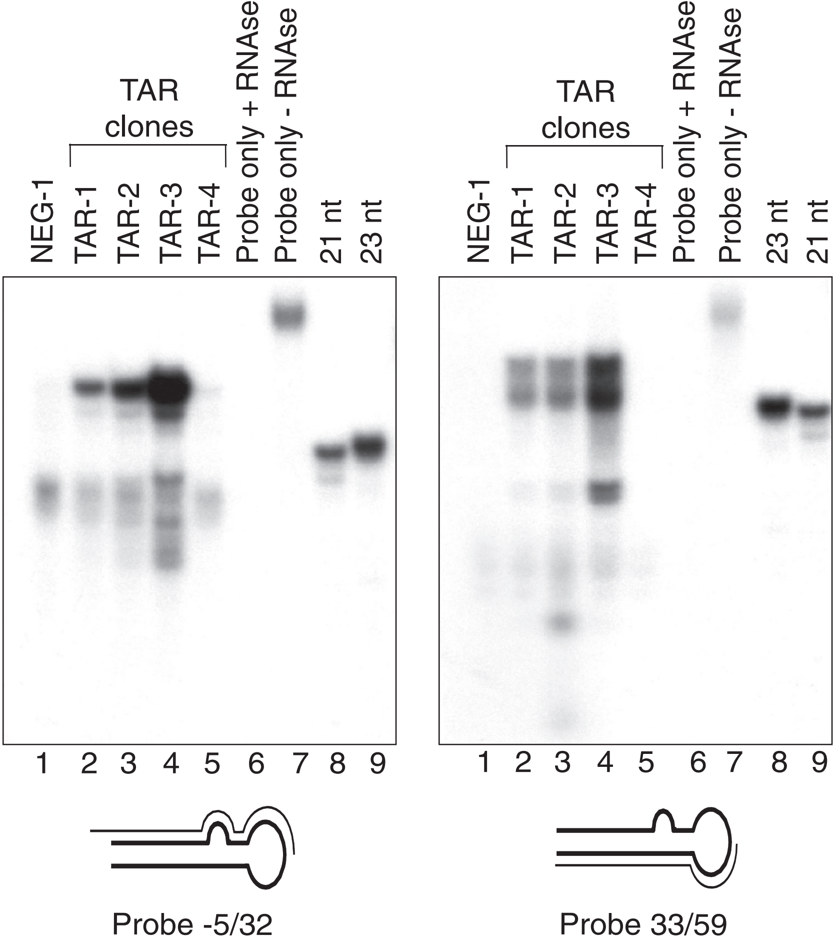

Additional File 2. Expression of TAR-derived miR-TAR-5p and miR-TAR-3p in four (4) different TAR-expressing Jurkat cell lines. Small RNAs (<200 nt) were isolated and HIV-1 miRNAs were detected by RPA using RNA probes directed against HIV-1 TAR nt -5/32 (left panel) and nt 33/64 (right panel) described previously [23]. The protected RNA species were visualized, in parallel to size markers, by denaturing PAGE and autoradiography.
