# Supplementary material for: Regulation of host gene expression by HIV-1 TAR microRNAs
Source: Retrovirology. 2013 Aug 12;10:86. doi: 10.1186/1742-4690-10-86 (PMC3751525; doi:10.1186/1742-4690-10-86)
Supplement: Additional file 3 — Cell cycle analysis for Jurkat TAR-expressing and NEG cell lines. Analysis of the cell cycle by flow cytometry in Jurkat TAR-expressing cell lines (Jurkat TAR 1, 2, 3 and 4) and in control cells (Jurkat WT and NEG-1) using propidium iodide (PI). [file 1742-4690-10-86-S3.pdf]

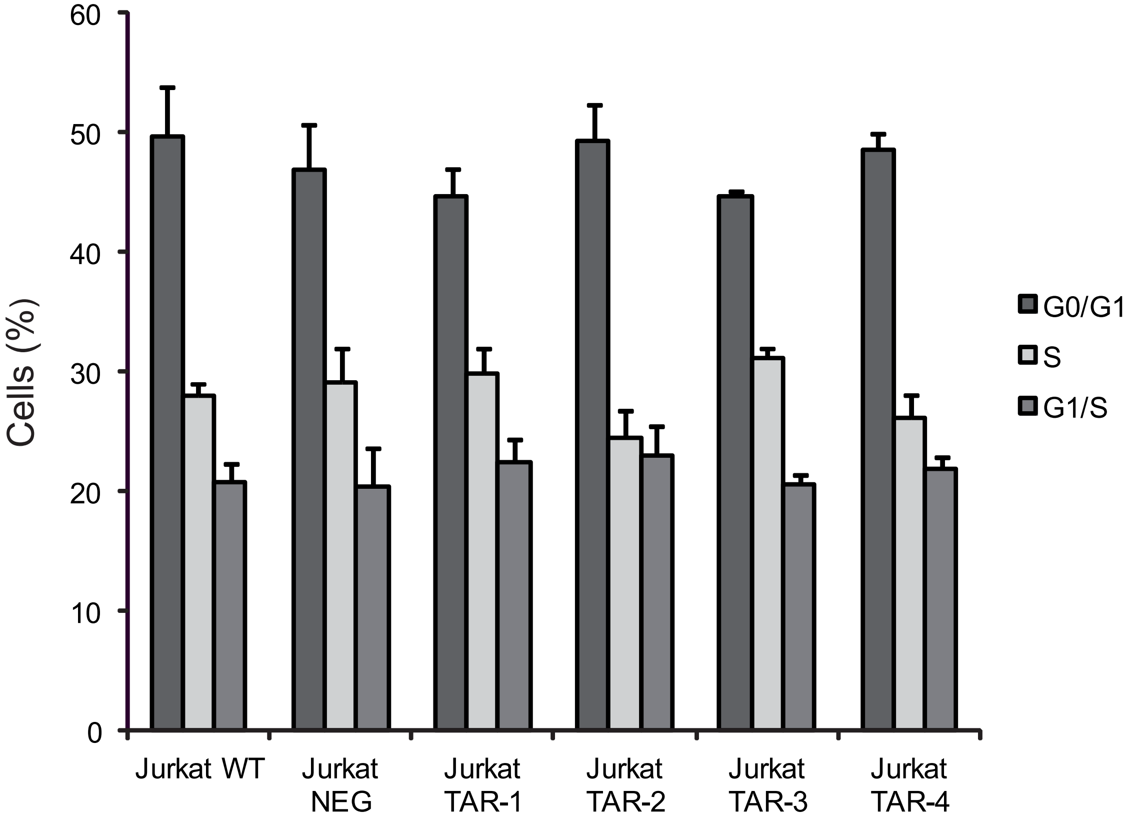


**Additional File 3. Cell cycle analysis of Jurkat TAR-expressing and NEG cell lines.** Cells (2x10E6) were resuspend in 0.5 ml PBS, mixing thoroughly to avoid clumps and 4.5 ml of ice-old 70% EtOH was added dropwise. Cells were kept on ice then stored at -20°C overnight. The next day, cells were centrifuge at 400 *g* for 5 minutes, EtOH was aspirate and cells were resuspended in 5 ml PBS 1X, 0.1% BSA. After another centrifugation, cells were resupended in 500 µl of propidium iodide (PI) staining solution (for 1 ml; 780 µl PBS, 0.1% Triton X-100, 20 µl PI at 1 mg/ml, 200 µl RNAse A at 1 mg/ml). Cells were kept at 4°C overnight and analyzed by flow cytometry the following day.
