# Supplementary material for: Regulation of host gene expression by HIV-1 TAR microRNAs
Source: Retrovirology. 2013 Aug 12;10:86. doi: 10.1186/1742-4690-10-86 (PMC3751525; doi:10.1186/1742-4690-10-86)
Supplement: Additional file 4 — NPM/B23 protein expression is downregulated by both miR-TAR-5p and miR-TAR-3p. Schematic, reporter assay and Western blots showing U6-sh5p and U6-sh3p downregulated NPM/B23 in HEK 293 cells. [file 1742-4690-10-86-S4.pdf]

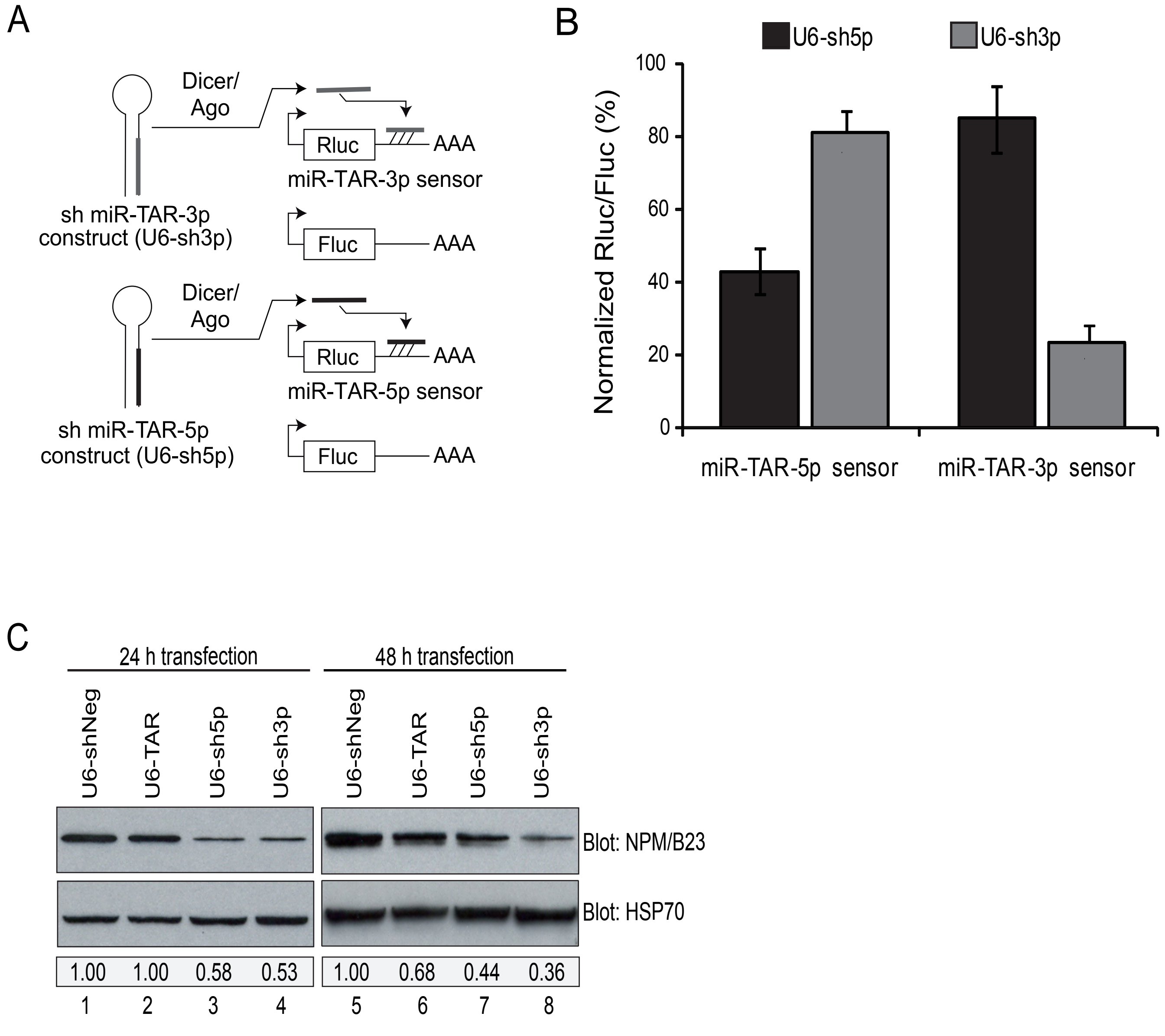

Additional File 4.NPM/B23 protein expression is downregulated by both miR-TAR-5p and miR-TAR-3p. A) Experimental approach aimed to assess TAR miRNAs expressed individually in a functional reporter gene activity assay. Both TAR miRNAs are expressed from a U6 promoter and are processed from a classical stem-loop hairpin. B) The miR-TAR-5p (U6-sh5p) or the miR-TAR-3p (U6-sh3p) or U6-sh-NEG (negative control) expression constructs (250 ng) were cotransfected with either miR-TAR-5p or miR-TAR-3p sensors for 24 h in HEK 293 cells. Renilla (Rluc) and Firefly (Fluc) luciferase were measu-red, and the Rluc/Fluc ratio was normalized on U6-NEG data. Results are expressed as mean ± s.e.m. (n = 5 experiments, in duplicate). C) Western blot analysis of NPM/B23 protein expression in HEK 293 cells transiently expressing U6-shNEG, U6-TAR, U6-sh5p or U6-sh3p RNA for 24 or 48 hours.
