# Supplementary material for: Regulation of host gene expression by HIV-1 TAR microRNAs
Source: Retrovirology. 2013 Aug 12;10:86. doi: 10.1186/1742-4690-10-86 (PMC3751525; doi:10.1186/1742-4690-10-86)
Supplement: Additional file 5 — miRTAR predictions for HIV-1 miR-TAR-5p targets. List of the messenger RNAs targeted by miR-TAR-5p, based on miRTAR predictions. [file 1742-4690-10-86-S5.pdf]

Additional File 5: miRTAR predictions for HIV-1 miR-TAR-5p targets

Gene ID	Gene symbol	Gene description	Nb of target sites
ENSG00000120049	KCNIP2	Kv channel interacting protein 2	9
ENSG00000010803	SCMH1	Sex comb on midleg homolog 1 (Drosophila)	8
ENSG00000155657	TTN	Titin	7
ENSG00000124508	BTN2A2	Butyrophilin, subfamily 2, member A2	6
ENSG00000100060	MFNG	MFNG O-fucosylpeptide 3-beta-N-acetylglucosaminyltransferase	6
ENSG00000114439	BBX	Bobby sox homolog (Drosophila)	4
ENSG00000160179	ABCG1	ATP-binding cassette, sub-family G	4
ENSG00000135596	MICAL1	Microtubule associated monoxygenase, calponin and LIM domain containing 1	4
ENSG00000036828	CASR	Calcium-sensing receptor	4
ENSG00000143847	PPFIA4	Protein tyrosine phosphatase, receptor type, f polypeptide (PTPRF), interacting protein (liprin), alpha 4	4
ENSG00000112294	ALDH5A1	Aldehyde dehydrogenase 5 family, member A1	4
ENSG00000072135	PTPN18	Protein tyrosine phosphatase, non-receptor type 18 (brain-derived)	4
ENSG00000138668	HNRNPD	Heterogeneous nuclear ribonucleoprotein D (AU-rich element RNA binding protein 1, 37kDa)	4
ENSG00000178038	ALS2CL	ALS2 C-terminal like	4
ENSG00000008853	RHOBTB2	Rho-related BTB domain containing 2	4
ENSG00000136237	RHOBTB2	Rap guanine nucleotide exchange factor (GEF) 5	4
ENSG00000020633	RUNX3	Runt-related transcription factor 3	4
ENSG00000160593	AMICA1	Adhesion molecule, interacts with CXADR antigen 1	4
ENSG00000019144	PHLDB1	Pleckstrin homology-like domain, family B, member 1	4
ENSG00000162543	UBXN10	UBX domain protein 10	4
ENSG00000166091	CMTM5	CKLF-like MARVEL transmembrane domain containing 5	4
ENSG00000131069	ACSS2	Acyl-CoA synthetase short-chain family member 2	4
ENSG00000124214	STAU1	Staufen, RNA binding protein, homolog 1 (Drosophila)	4
ENSG00000072210	ALDH3A2	Aldehyde dehydrogenase 3 family, member A2	4
ENSG00000187122	SLIT1	Slit homolog 1 (Drosophila)	3
ENSG00000187848	P2RX2	Purinergic receptor P2X, ligand-gated ion channel, 2	3
ENSG00000011304	PTB1	Polypyrimidine tract binding protein 1	3
ENSG00000101460	MAP1LC3A	Microtubule-associated protein 1 light chain 3	3

		alpha	
ENSG00000151136	BTBD11	BTB (POZ) domain containing 11	3
ENSG00000090857	PDPR	Pyruvate dehydrogenase phosphatase regulatory subunit	3
ENSG00000130695	CEP85	Centrosomal protein 85kDa	3
ENSG00000185811	IKZF1	IKAROS family zinc finger 1 (Ikaros)	3
ENSG00000132854	KANK4	KN motif and ankyrin repeat domains 4	3
ENSG00000197891	SLC22A12	Solute carrier family 22 (organic anion/urate transporter), member 12	3
ENSG00000167733	HSD11B1L	Hydroxysteroid (11-beta) dehydrogenase 1-like	3
ENSG00000107521	HPS1	Hermansky-Pudlak syndrome 1	3
ENSG00000133961	NUMB	Numb homolog (Drosophila)	3
ENSG00000125618	PAX8	Paired box 8	3
ENSG00000105327	BBC3	BCL2 binding component 3	3
ENSG00000124205	EDN3	Endothelin	3
ENSG00000112033	PPARD	Peroxisome proliferator-activated receptor delta	3
ENSG00000144115	THNSL2	Threonine synthase-like 2 (S. cerevisiae)	3
ENSG00000002745	WNT16	Wingless-type MMTV integration site family, member 16	3
ENSG00000148343	FAM73B	Family with sequence similarity 73, member B	3
