# Supplementary material for: Regulation of host gene expression by HIV-1 TAR microRNAs
Source: Retrovirology. 2013 Aug 12;10:86. doi: 10.1186/1742-4690-10-86 (PMC3751525; doi:10.1186/1742-4690-10-86)
Supplement: Additional file 6 — miRTAR predictions for HIV-1 miR-TAR-3p targets. List of the messenger RNAs targeted by miR-TAR-3p, based on miRTAR predictions. [file 1742-4690-10-86-S6.pdf]

Additional File 6: miRTAR predictions for HIV-1 miR-TAR-3p targets.

Ensembl Gene ID	Gene symbol	Gene description	Nb of target sites
ENSG00000127554	GFER	Growth factor, augmenter of liver regeneration	12
ENSG00000068028	RASSF1	Ras association (RalGDS/AF-6) domain family member 1	12
ENSG00000186575	NF2	Neurofibromin 2 (merlin)	12
ENSG00000026508	CD44	CD44 molecule (Indian blood group)	10
ENSG00000126091	ST3GAL3	ST3 beta-galactoside alpha-2,3-sialyltransferase 3	9
ENSG00000120049	KCNIP2	Kv channel interacting protein 2	9
ENSG00000103034	NDRG4	NDRG family member 4	8
ENSG00000166091	CMTM5	CKLF-like MARVEL transmembrane domain containing 5	8
ENSG00000187122	SLIT1	Slit homolog 1 (Drosophila)	8
ENSG00000103549	RNF40	Ring finger protein 40, E3 ubiquitin protein ligase	7
ENSG00000065534	MYLK	Myosin light chain kinase	6
ENSG00000064012	CASP8	Caspase 8, apoptosis-related cysteine peptidase	6
ENSG00000142961	MOB3C	MOB kinase activator 3C	6
ENSG00000100403	ZC3H7B	Zinc finger CCCH-type containing 7B	6
ENSG00000161405	IKZF3	IKAROS family zinc finger 3 (Aiolos)	6
ENSG00000124920	C11orf9	Chromosome 11 open reading frame 9	6
ENSG00000153303	FRMD1	FERM domain containing 1	6
ENSG00000173698	GPR64	G-protein-coupled receptor 64	6
ENSG00000165246	NLGN4Y	Neuroligin 4, Y-linked	6
ENSG00000135924	DNAJB2	DnaJ (Hsp40) homolog, subfamily B, member 2	6
ENSG00000204248	COL11A2	Collagen, type XI, alpha 2	6
ENSG00000184985	SORCS2	Sortilin-related VPS10 domain containing receptor 2	6
ENSG00000112977	DAP	Death-associated protein	5
ENSG00000088367	EPB41L1	Erythrocyte membrane protein band 4.1-like 1	5
ENSG00000188878	FBF1	Fas (TNFRSF6) binding factor 1	5
ENSG00000143537	ADAM15	ADAM metallopeptidase domain 15	5
ENSG00000105227	PRX	Periaxin	5
ENSG00000167094	TTC16	Tetratricopeptide repeat domain 16	5
ENSG00000081853	PCDHGA2	Protocadherin gamma subfamily A, 2	5
ENSG00000010165	METTL13	Methyltransferase like 13	4
ENSG00000197565	COL4A6	Collagen, type IV, alpha 6	4
ENSG00000198055	GRK6	G-protein-coupled receptor kinase 6	4
ENSG00000131069	ACSS2	Acyl-CoA synthetase short-chain family member 2	4
ENSG00000072135	PTPN18	Protein tyrosine phosphatase, non-receptor	4

		type 18 (brain-derived)	
ENSG00000198053	SIRPA	Signal-regulatory protein alpha	4
ENSG00000171596	NMUR1	Neuromedin U receptor 1	4
ENSG00000196821	C6orf106	Chromosome 6 open reading frame 106	4
ENSG00000125510	OPRL1	Opiate receptor-like 1	4
ENSG00000183495	EP400	E1A binding protein p400	4
ENSG00000159173	TNNI1	Troponin I type 1 (skeletal, slow)	4
ENSG00000166448	TMEM130	Transmembrane protein 130	4
ENSG00000185504	C17orf170	Chromosome 17 open reading frame 70	4
ENSG00000124214	STAU1	Staufen, RNA binding protein, homolog 1 (Drosophila)	4
ENSG00000197558	SSPO	SCO-spondin homolog (Bos taurus)	4
ENSG00000198742	SMURF1	SMAD specific E3 ubiquitin protein ligase 1	4
ENSG00000196632	WNK3	WNK lysine deficient protein kinase 3	4
ENSG00000100060	MFNG	MFNG O-fucosylpeptide 3-beta-N-acetylglucosaminyltransferase	4
ENSG00000082458	DLG3	Discs, large homolog 3 (Drosophila)	4
ENSG00000159842	ABR	Active BCR-related	4
ENSG00000019144	PHLDB1	Pleckstrin homology-like domain, family B, member 1	4
ENSG00000175785	PRIMA1	Proline rich membrane anchor 1	4
ENSG00000197818	SLC9A8	Solute carrier family 9, subfamily A (NHE8, cation proton antiporter 8), member 8	4
ENSG00000178752	FAM132B	Family with sequence similarity 132, member B	4
ENSG00000077279	DCX	Doublecortin	4
ENSG00000169436	COL22A1	Collagen, type XXII, alpha 1	4
ENSG00000112319	EYA4	Eyes absent homolog 4 (Drosophila)	4
ENSG00000080189	SLC35C2	Solute carrier family 35, member C2	4
ENSG00000159023	EPB41	Erythrocyte membrane protein band 4.1	4
ENSG00000090975	PITPNM2	Phosphatidylinositol transfer protein, membrane-associated 2	4
ENSG00000165480	SKA3	Spindle and kinetochore associated complex subunit 3	4
ENSG00000141753	IGFBP4	Insulin-like growth factor binding protein 4	3
ENSG00000127191	TRAF2	TNF receptor-associated factor 2	3
ENSG00000166166	TRMT61A	tRNA methyltransferase 61 homolog A (S. cerevisiae)	3
ENSG00000116604	MEF2D	Myocyte enhancer factor 2D	3
ENSG00000185340	GAS2L1	Growth arrest-specific 2 like 1	3
ENSG00000073417	PDE8A	Phosphodiesterase 8A	3
ENSG00000119689	DLST	Dihydrolipoamide S-succinyltransferase (E2 component of 2-oxo-glutarate complex)	3
ENSG00000106336	FBX024	F-box protein 24	3
ENSG00000100083	GGA1	Golgi-associated, gamma adaptin ear containing, ARF binding protein 1	3
ENSG00000068078	FGFR3	Fibroblast growth factor receptor 3	3
ENSG00000151136	BTBD11	BTB (POZ) domain containing 11	3
ENSG00000105327	BBC3	BCL2 binding component 3	3

ENSG00000131067	GGT7	Gamma-glutamyltransferase 7	3
ENSG00000182841	RRP7B	Ribosomal RNA processing 7 homolog B (S. cerevisiae)	3
ENSG00000187848	P2RX2	Purinergic receptor P2X, ligand-gated ion channel, 2	3
ENSG00000119946	CNNM1	Cyclin M1	3
ENSG00000166444	ST5	Suppression of tumorigenicity 5	3
ENSG00000133460	SLC2A11	Solute carrier family 2 (facilitated glucose transporter), member 11	3
ENSG00000102858	MGRN1	Mahogunin ring finger 1, E3 ubiquitin protein ligase	3
ENSG00000186111	PIP5K1C	Phosphatidylinositol-4-phosphate 5-kinase, type I, gamma	3
ENSG00000058668	ATP2B4	ATPase, Ca++ transporting, plasma membrane 4	3
ENSG00000119720	C14orf102	Chromosome 14 open reading frame 102	3
ENSG00000141084	RANBP10	RAN binding protein 10	3
ENSG00000151067	CACNA1C	Calcium channel, voltage-dependent, L type, alpha 1C subunit	3
ENSG00000124225	PMEPA1	Prostate transmembrane protein, androgen induced 1	3
ENSG00000198624	CCDC69	Coiled-coil domain containing 69	3
ENSG00000002919	SNX11	Sorting nexin 11	3
ENSG00000160953	MUM1	Melanoma associated antigen (mutated) 1	3
ENSG00000163754	GYG1	Glycogenin 1	3
ENSG00000112561	TFEB	Transcription factor EB	3
ENSG00000105662	CRTC1	CREB regulated transcription coactivator 1	3
ENSG00000168395	ING5	Inhibitor of growth family, member 5	3
ENSG00000143776	CDC42BPA	CDC42 binding protein kinase alpha (DMPK-like)	3
ENSG00000090539	CHRD	Chordin	3
ENSG00000137409	MTCH1	Mitochondrial carrier 1	3
ENSG00000173918	C1QTNF1	C1q and tumor necrosis factor related protein 1	3
ENSG00000079805	DNM2	Dynamin 2	3
ENSG00000146054	TRIM7	Tripartite motif containing 7	3
ENSG00000117461	PIK3R3	Phosphoinositide-3-kinase, regulatory subunit 3 (gamma)	3
ENSG00000114779	ABHD14B	Abhydrolase domain containing 14B	3
ENSG00000107521	HPS1	Hermansky-Pudlak syndrome 1	3
ENSG00000134874	DZIP1	DAZ interacting protein 1	3
ENSG00000104731	KLHDC4	Kelch domain containing 4	3
ENSG00000115163	CENPA	Centromere protein A	3
ENSG00000127952	STYXL1	Serine/threonine/tyrosine interacting-like 1	3
ENSG00000149582	TMEM25	Transmembrane protein 25	3
ENSG00000197586	ENTPD6	Ectonucleoside triphosphate diphosphohydrolase 6 (putative)	3

ENSG00000124191	TOX2	TOX high mobility group box family member 2	3
ENSG00000186807	ANXA8L2	Annexin A8-like 2	3
ENSG00000182473	EXOC7	Exocyst complex component 7	3
