# Supplementary material for: Regulation of host gene expression by HIV-1 TAR microRNAs
Source: Retrovirology. 2013 Aug 12;10:86. doi: 10.1186/1742-4690-10-86 (PMC3751525; doi:10.1186/1742-4690-10-86)
Supplement: Additional file 7 — Both miR-TAR-5p and miR-TAR-3p regulate gene expression through the 3’UTR of NPM/B23. Reporter gene assay showing the regulation of NPM/B23 5’UTR, ORF and 3’UTR by the miR-TAR-5p or miR-TAR-3p, when expressed individually from conventional stem-loop. [file 1742-4690-10-86-S7.pdf]

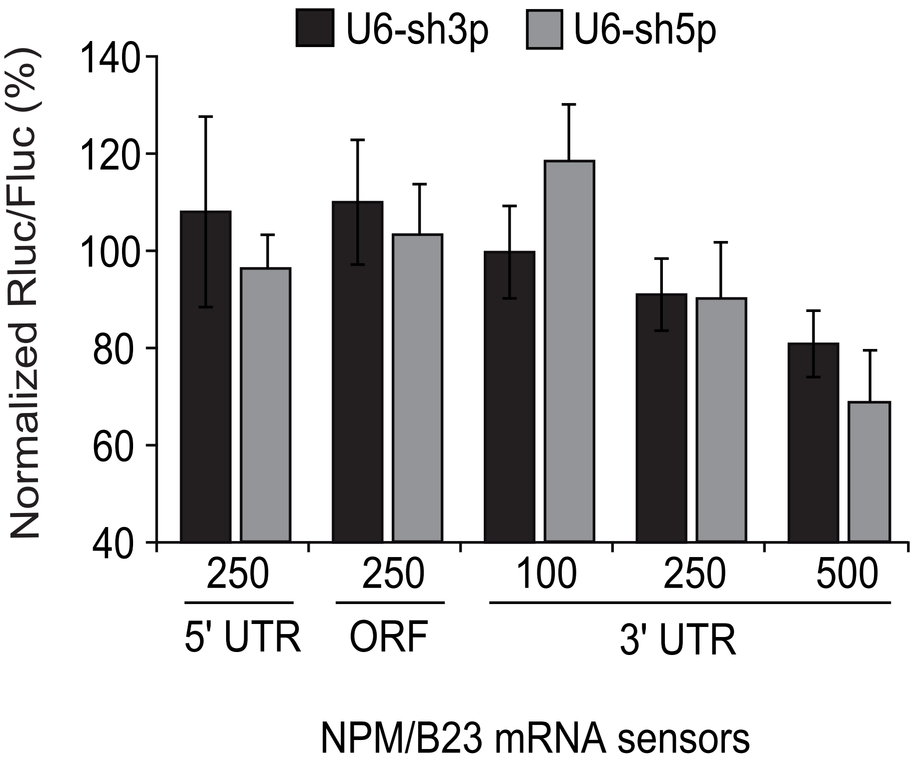

Additional File 7. Both miR-TAR-5p and miR-TAR-3p regulate gene expression through the 3'UTR of NPM/B23. HEK 293 cells were transiently transfected for 48 hours with a construct encoding either miR-TAR-5p or miR-TAR-3p and a reporter construct in which Rluc ORF is coupled with either the 5'UTR, ORF or 3'UTR of NPM/B23 mRNA. Renilla (Rluc) and Firefly (Fluc) luciferase were measured, and Rluc/Fluc ratios were calculated and normalized on psiNEG data. Results are expressed as mean ± s.e.m. (n= 4 to 6 experiments, in duplicate).
