# Supplementary material for: Regulation of host gene expression by HIV-1 TAR microRNAs
Source: Retrovirology. 2013 Aug 12;10:86. doi: 10.1186/1742-4690-10-86 (PMC3751525; doi:10.1186/1742-4690-10-86)
Supplement: Additional file 8 — Drug stimulation of J-Lat cell lines. Flow cytometry analysis of J-Lat clones 6.3, 8.4, 9.2 and 10.6 stimulated with TNF-α, PMA, prostatin, TSA, SAHA, valproic acid and HMBA for 18 hours. [file 1742-4690-10-86-S8.pdf]

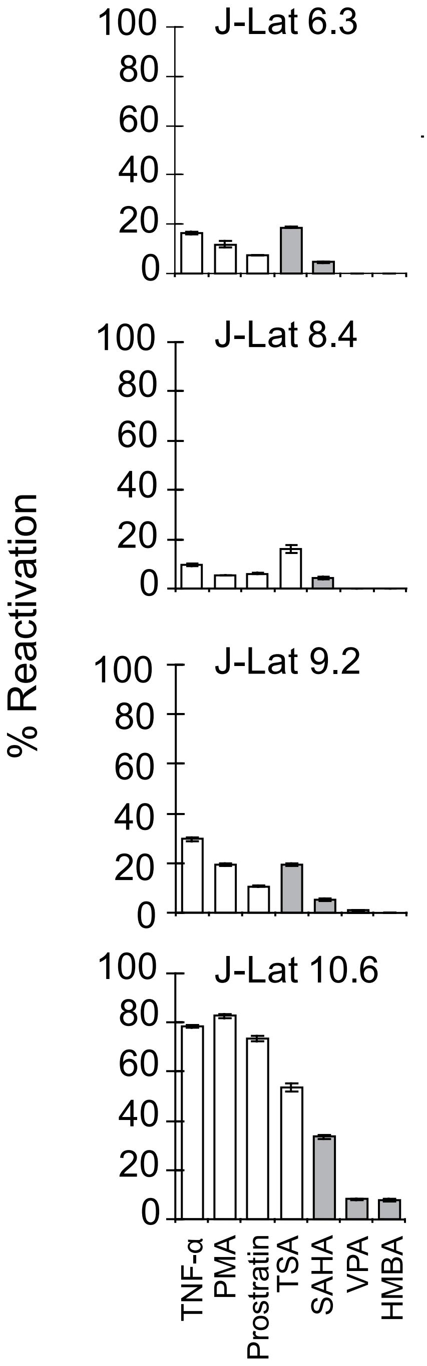

Additional File 8. Drug stimulation of J-Lat clones. J-Lat clones were stimulated with the following: tumor necrosis factor alpha (TNF-α), phorbol 12-myristate 13-acetate (PMA), prostratin (white bars, columns 1-3) or Trichostatin A (TSA), suberoylanilide hydroxamic acid (SAHA), valproic acid, and hexamethylene bisacetamide (HMBA) (gray bars, columns 4-7). Flow cytometry measurements were performed 18 hours after stimulation to measure the change in the fraction of latent cells, as indicated by % reactivation (right panel). All data are averages of biological triplicates, and error bars are standard deviations.
