# Supplementary material for: Regulation of host gene expression by HIV-1 TAR microRNAs
Source: Retrovirology. 2013 Aug 12;10:86. doi: 10.1186/1742-4690-10-86 (PMC3751525; doi:10.1186/1742-4690-10-86)
Supplement: Additional file 9 — Oligonucleotide sequences. List of oligonucleotide sequences used for cloning, PCR and as DNA probes. [file 1742-4690-10-86-S9.pdf]

Additional File 9. Oligonucleotide sequences.

Names	Sequences (5'-3')
Caspase 8 3'UTR For	GCGCGCCTCGAGTGGTGCTATTTTGTTTGTTTTG
Caspase 8 3'UTR Rev	GCGCGCGCGGCCGCTAAGATCAAATTATATTTTATTC
Aiolos 3'UTR For	GCGCGCCTCGAGATATCTGGTCTCAGGGATTGCTCCTATGTATTC AGC
Aiolos 3'UTR Rev	GCGACGCGCGCGCGCGGCCGCTTTGTTTTCTGTTGGTGCTGCC
Ikaros 3'UTR For	GCGCGCCTCGAGAGCCCTCCCGCGCCCCCACCCCAGACCCCGA GC
Ikaros 3'UTR Rev	GCGCGCGCGCGCGCGGCCGCAAAGCCCTTGATCCTCTGTCTCTC TTCC
NPM/B23 3'UTR For	GCGCGCCTCGAGGAAAATAGTTTAAACAATTTG
NPM/B23 3'UTR Rev	GCATGCATGCATGCGGCCGCAAAATACTGAGTTTTATTTCAC
NPM/B23 5'UTR For	GCGCGCCTCGAGGGGAAGCGCTCGCGAGATC
NPM/B23 5'UTR Rev	GCATGCATGCATGCGGCCGCCGGGTGGCGGCACGCACTTAGG
NPM/B23 ORF For	GCGCGCCTCGAGGAAGATTCGATGGACATGG
NPM/B23 ORF Rev	GCATGCATGCATGCGGCCGCAAGAGACTTCCTCCACTGCC
Caspase 8 qPCR For	GCCAGGAAAGGGTGGAGCGG
Caspase 8 qPCR Rev	TGAGCCCTGCCTGGTGTCTGA
Aiolos qPCR For	GCACAAGGAGCGCTGCCGTA
Aiolos qPCR Rev	GTGTCTGGACCAAGGGGCGC
Ikaros qPCR For	GCCACACTGGAGAACGGCCC
Ikaros qPCR Rev	GCCCGGAAGGCCCATGCTTT
NPM/B23 qPCR For	TTGTTGAAGCAGAGGCAATG
NPM/B23 qPCR Rev	AATATGCACTGGCCCTGAAC
GAPDH qPCR For	GCCTTCCGTGTCCCCACTGC
GAPDH qPCR Rev	CAATGCCAGCCCCAGCGTCA
Tubulin qPCR For	TCTTCAGTGAGACGGGGG
Tubulin qPCR Rev	GTGATGAGTTGCTCAGGG
sh5p up	ACCGCTGGTCTAACCAGAGAGACTTCCTGTCATCTCTCTGGTTAG ACCAGCTTTTTC
sh5p down	TGCAGAAAAAGCTGGTCTAACCAGAGAGATGACAGGAAGTCTCTC TGGTTAGACCAG
sh3p up	ACCGTCCCTAGTTAGCCAGAGACTTCCTGTCATCTCTGGCTAACT AGGGACTTTTTC
sh3p down	TGCAGAAAAAGTCCCTAGTTAGCCAGAGATGACAGGAAGTCTCTG GCTAACTAGGGA
NEG-3p probe	GTATAATACACCGCGCTACTG
miR-TAR-3p probe	GTCCCTAGTTAGCCAGAGATG
U6 probe	TATGGAACGCTTCTCGAATT
